# Invasion by the Alien Tree *Prunus serotina* Alters Ecosystem Functions in a Temperate Deciduous Forest

**DOI:** 10.3389/fpls.2017.00179

**Published:** 2017-02-14

**Authors:** Raf Aerts, Michael Ewald, Manuel Nicolas, Jérôme Piat, Sandra Skowronek, Jonathan Lenoir, Tarek Hattab, Carol X. Garzón-López, Hannes Feilhauer, Sebastian Schmidtlein, Duccio Rocchini, Guillaume Decocq, Ben Somers, Ruben Van De Kerchove, Karolien Denef, Olivier Honnay

**Affiliations:** ^1^Ecology, Evolution and Biodiversity Conservation Section, KU LeuvenLeuven, Belgium; ^2^Division Forest, Nature and Landscape, KU LeuvenLeuven, Belgium; ^3^Institut für Geographie und Geoökologie, Karlsruher Institut für TechnologieKarlsruhe, Germany; ^4^Office National des Forêts, Fontainebleau and CompiègneFrance; ^5^Geography, University of Erlangen-NürnbergErlangen, Germany; ^6^Ecologie et Dynamique des Systèmes Anthropisés (EDYSAN, FRE 3498 CNRS-UPJV), Université de Picardie Jules VerneAmiens, France; ^7^Department of Biodiversity and Molecular Ecology, Research and Innovation Centre (CRI) - Edmund Mach FoundationTrento, Italy; ^8^Unit Remote Sensing and Earth Observation Processes, VITO NVMol, Belgium; ^9^Central Instrument Facility, Department of Chemistry, Colorado State UniversityFort Collins, CO, USA

**Keywords:** American black cherry, biological invasion, biogeochemical cycles, canopy foliar nutrients, heterotrophic respiration, litter, exotic species, invasive species

## Abstract

Alien invasive species can affect large areas, often with wide-ranging impacts on ecosystem structure, function, and services. *Prunus serotina* is a widespread invader of European temperate forests, where it tends to form homogeneous stands and limits recruitment of indigenous trees. We hypotesized that invasion by *P. serotina* would be reflected in the nutrient contents of the native species' leaves and in the respiration of invaded plots as efficient resource uptake and changes in nutrient cycling by *P. serotina* probably underly its aggressive invasiveness. We combined data from 48 field plots in the forest of Compiègne, France, and data from an experiment using 96 microcosms derived from those field plots. We used general linear models to separate effects of invasion by *P. serotina* on heterotrophic soil and litter respiration rates and on canopy foliar nutrient content from effects of soil chemical properties, litter quantity, litter species composition, and tree species composition. In invaded stands, average respiration rates were 5.6% higher for soil (without litter) and 32% higher for soil and litter combined. Compared to indigenous tree species, *P. serotina* exhibited higher foliar N (+24.0%), foliar P (+50.7%), and lower foliar C:N (−22.4%) and N:P (−10.1%) ratios. *P. serotina* affected foliar nutrient contents of co-occuring indigenous tree species leading to decreased foliar N (−8.7 %) and increased C:N ratio (+9.5%) in *Fagus sylvatica*, decreased foliar N:P ratio in *Carpinus betulus* (−13.5%) and *F. sylvatica* (−11.8%), and increased foliar P in *Pinus sylvestris* (+12.3%) in invaded vs. uninvaded stands. Our results suggest that *P. serotina* is changing nitrogen, phosphorus, and carbon cycles to its own advantage, hereby increasing carbon turnover via labile litter, affecting the relative nutrient contents in the overstory leaves, and potentially altering the photosynthetic capacity of the long-lived indigenous broadleaved species. Uncontrolled invasion of European temperate forests by *P. serotina* may affect the climate change mitigation potential of these forests in the long term, through additive effects on local nutrient cycles.

## Introduction

Alien invasive plant species have important effects on the diversity and stability of ecosystems (Ehrenfeld, 2003; Hejda et al., 2009). Alien invasive species may replace native biodiversity, usually by affecting the regeneration of native species and suppressing native plant growth (Belnap et al., 2005). Once established in high abundance, alien invasive species may exert a broad range of impacts on the structure and function of ecosystems and their ecosystem services (Vilà et al., 2010, 2011). Alien invasive species can, for instance, affect belowground carbon pools (Liao et al., 2007; Kramer et al., 2012) or change decomposition rates via impacts on litter quality (Ashton et al., 2005; Zhang et al., 2014, 2016), hereby potentially altering the ecosystems' environmental conditions and indirectly driving changes in plant community composition (Halarewicz and Żołnierz, 2014). Alien invasive species may also compete for pollinator species and have negative effects on the reproductive output of native plant species (Thijs et al., 2012). Alien invasive plant species may even have direct impacts on human health, for instance by producing allergenic pollen that exacerbate respiratory diseases (Wayne et al., 2002; Richter et al., 2013).

In forests, alien invasive species often affect large areas and their impacts on plant communities, soil microbiota and litter quality may accelerate or decelerate local nutrient cycles (Ehrenfeld, 2003; Lazzaro et al., 2014), promoting losses or gains in local nutrient stocks (Kramer et al., 2012). Such impacts potentially have wide-ranging consequences, for instance when alien invasive species increase greenhouse gas emissions from the soil (Chen et al., 2015). It is important to test the impacts of invasive plant species on ecosystem functioning, because this information and data on landscape characteristics that enable or limit plant invasions (Chabrerie et al., 2007), are needed for establishing management plans for the invasive species, including plans and methods to prevent, detect, and eradicate invasive alien species (Hulme et al., 2009; Chytrý et al., 2011). To reliably support decisions based on impact, this information needs to be species and site specific (Hejda et al., 2009).

American black cherry (*Prunus serotina* Ehrh., Rosaceae) is a widespread invader of European temperate forests. Native to North America, *P. serotina* was introduced in Western Europe as an ornamental plant and as a timber species. Later it was repeatedly planted in high densities in the understory of plantations to improve their litter quality or to reduce fire risk (Starfinger et al., 2003; Lorenz et al., 2004). Its shade-tolerant seedlings and saplings persist in the understory of newly colonized forest patches and become light-demanding, fast-growing and early reproducing trees once a gap is created (Godefroid et al., 2005; Closset-Kopp et al., 2007). Being able to sprout vigorously from its roots, *P. serotina* tends to form dense, homogeneous stands with a high leaf area index (LAI; Urban et al., 2009), overshadowing seedlings and limiting recruitment of indigenous trees (Vanhellemont et al., 2010). The altered environmental conditions following invasion by *P. serotina* also have negative effects on native understory biodiversity (Verheyen et al., 2007), for instance by causing a functional shift toward shade-tolerant, short-living ruderals (Chabrerie et al., 2010), and species with high nutrient demand (Halarewicz and Żołnierz, 2014). Other effects of the invasion of forest stands by *P. serotina* include changes in aboveground biomass (e.g., higher biomass in invaded stands; Dassonville et al., 2008) and humus quality (e.g., improvement of humus quality in pine forests; Lorenz et al., 2004) but as with other exotic species, these effects are highly site-specific (Koutika et al., 2007) and may also depend on other (human) disturbances in the forest (Halarewicz and Żołnierz, 2014). As eradication of *P. serotina* is notoriously difficult and usually requires the application of contested herbicides (Klotz, 2007), it has been argued that it may be more useful to accept and manage rather than to try to eradicate the species (Starfinger et al., 2003). For instance, *P. serotina* has been included as a useful species in the silvicultural cycle to prevent the leaching of nitrate to the groundwater (Lorenz et al., 2004). Other silvicultural measures to control *P. serotina* include removing seed trees or trees with larger diameter, enhancing structural diversity or increasing the proportion of shade-tolerant species (Buysse, 2012; Sitzia et al., 2016).

Here we quantify the ecosystem-level impacts of *P. serotina* invading a European temperate forest to assess the potential long-term consequences of uncontrolled invasion. As *P. serotina* is an aggressive and fast-growing invader (Closset-Kopp et al., 2007) that is probably more efficient in capturing resources than native species (Dassonville et al., 2008), we hypotesized that invasion by *P. serotina* would be reflected in the nutrient contents of the native species' leaves and in the respiration of invaded plots. We are interested in the leaf chemistry of the indigenous species as it may reflect impacts of invasive species on the native species' photosynthetic capacity and, subsequently, net primary productivity (Hikosaka et al., 2016). We are also interested in respiration as it reflects impacts of invasion on carbon turnover (Zhang et al., 2014). Combined, our results may help us to understand how invasion by *P. serotina* affects productivity, carbon sequestration and climate regulation of European temperate forests. To test our hypothesis, we combined data from 48 field plots and data from an experiment using 96 microcosms derived from those field plots. We used general linear models to separate invasion effects from effects of soil chemical properties, litter quantity, litter species composition and tree species composition. Specifically, we assessed the impact of *P. serotina* on heterotrophic respiration from forest soil and litter, and on the carbon, nitrogen and phosphorus content in the leaves of co-occurring indigenous trees.

## Materials and methods

### Study location

The study was performed in the temperate deciduous forest of Compiègne, located ~60 km north of Paris, in the Picardy region, northern France (49°22′N, 2°54′E; 32–152 m elevation; 677 mm mean annual precipitation, 10°C mean annual temperature, Chabrerie et al., 2008). The dominant soils are leached brown soils (luvisols, cambisols) but, locally, podzolised soils (podzols) and calcareous soils (leptosols) also occur. The soils developed in sedimentary substrates (palaeogeneous sands, cretaceous chalk sand, and limestone) variably covered by quaternary loess or alluvial deposits (Closset-Kopp et al., 2007). The forest, with a total surface area of ca. 15,000 ha, is mostly intensively managed as even-aged high forest. The dominant tree species are European beech (*Fagus sylvatica* L.), oaks [*Quercus robur* L. and *Q. petraea* (Matt.) Liebl.], European hornbeam (*Carpinus betulus* L.) and, on sandy soils, Scots pine (*Pinus sylvestris* L.). The silvicultural management units (stands) are homogeneous or mixed species even-aged stands, each stand covering on average (± SE mean) 13.34 ± 0.14 ha. The prevailing silvicultural systems are clearcutting and seed-tree shelterwood, in cycles of 100–220 years with thinning once every 4–15 years (Closset-Kopp et al., 2007; Chabrerie et al., 2008). The changes in light environment associated with clearcutting and a number of severe storm events have left ample opportunity for the rapid and widespread invasion of gaps by *P. serotina* (Closset-Kopp et al., 2007). It was locally reported as an invasive species for the first time in the early 1970's. Since then, it has spread over the entire forest and is present in more than half of the total area of the forest (Chabrerie et al., 2008).

### Sampling design

Using available distribution maps of *P. serotina* and examining published data from 64 plots of 25 × 25 m in the study area (Chabrerie et al., 2008), we stratified the forest in invaded and uninvaded stands and randomly selected 44 of the 64 existing plots for further study (see Supplementary Material for the protocol and illustrations of the respiration experiment). We established six additional plots at intermediate *P. serotina* invasion stage (both tree and shrub cover of *P. serotina* ≈30%; Chabrerie et al., 2008). Following our own field surveys of 2014, we omitted two plots due to partial missing data, thus totalizing 48 plots. Furthermore, we had to reclassify the plots as the invasion by *P. serotina* had noticeably progressed. We defined plots as invaded by *P. serotina* if the species' basal area exceeded the threshold of 0.05 m^2^ ha^−1^. By applying this threshold instead of using pure *P. serotina* presence data we avoided misclassifying plots that were only in a very early stage of invasion (very few seedlings present only). The final sampling design comprised 20 uninvaded plots and 28 invaded plots with basal areas of *P. serotina* ranging between 0.09 and 14.38 m^2^ ha^−1^. The selected plots were on average separated by 543 m (SE 42 m) from their nearest neighbor (distance between centroids) and were well-distributed throughout the forest (see Supplementary Material for a Google Earth Datafile containing the locations of the sample plots).

### Data collection

In each 25 × 25 m plot we recorded vegetation data and collected soil, litter and canopy leaf samples. For all live trees (woody species > 6 m tall) and shrubs (woody species between 2 and 6 m tall) we recorded species, girth at breast height (GBH; m) and the proportion (PR; %) of their vertical crown projection overlapping the plot.

We collected soil samples by pooling a number of subsamples gathered in each plot (“individual soil samples” sensu Reinhart and Rinella, 2016). A first sample comprised 10 soil cores which were 15-cm deep (without litter (L) and ectorganic OF horizon) obtained from 10 random locations within the plot; this sample was used for soil chemical analysis. A second sample comprised five analogous soil cores and was used for the respiration experiment. Similarly, a litter sample (L and OF horizons) was composed from four subsamples from random 0.25 × 0.25 m subplots. Soil and litter samples were air-dried. The air-dried soil samples for the decomposition experiment were stored at 1°C awaiting further analysis. Soil samples for chemical analysis and litter samples were oven-dried (soil: 72 h at 40°C; litter: 72 h at 60°C). The oven-dried litter samples were weighed and the available litter on the forest floor was then quantified as tons dry litter per ha. Soil and litter samples were collected in July 2014.

We used Model 55 Goose Guns (Marlin Firearms, Madison, NC) and a fully choked Select Sporting II 12M 12-gauge shotgun (Winchester Repeating Arms, Morgan, UT) with Buckshot 27 ammunition (27 × 6.2 mm lead pellets) to shoot 325 sets of leaf samples from 307 individual trees in 48 plots (see Supplementary Material for a video). We used the leaf sampling by shotgun method for a number of reasons: tree trunk climbing was not permitted in the forest; leaf sampling by shotgun is the standard method in the protocol of the French National Network for Long-term Forest Ecosystem Monitoring (RENECOFOR); and leaf sampling by shotgun was found to be much more time- and cost-efficient than any other protocol, including those that use boom trucks or throw-line launchers (see e.g., Youngentob et al., 2016). In each plot we aimed to collect leaves from three individuals per species. For broadleaved tree species, one set of 10–15 undamaged leaves was collected from whole branches or twigs shot down from the upper, sun-exposed part of the crown. For *P. sylvestris* two sets of needles were collected per tree: one from new shoots (year *N*) and one from 1-year-old shoots (year *N*−1). Each set of leaves was put in a labeled paper bag. Paper bags with leaf samples were put in plastic zip-loc bags per species and per plot and stored in a cooler. The leaf samples were oven-dried (48 h at 80°C). Canopy leaf samples were collected in July 2015.

### Soil and leaf analysis

The soil samples were sieved (<2 mm) to remove stones and root biomass. For each sample for the chemical analysis, we determined the average of two measurements of soil potential acidity using a NH_4_Cl extraction solution and a glass electrode; soil nitrogen (wt%) using a C/N-analyzer (TruSpec CN, LECO Corporation, St Joseph, MI, USA); soil phosphorus (mg kg^−1^) using the lactate extractable phosphate method; and concentrations of exchangeable bases Ca, K, Mg, and Na (cmol kg^−1^) by extraction with 1M NH_4_Cl. Soil organic matter content (SOM; wt%) was determined for a subsample of the soil collected for the respiration experiment by using the loss on ignition method in a muffle furnace (Hoogsteen et al., 2015).

Oven-dried leaf samples were milled prior to chemical analysis. Foliar carbon (C) (g kg^−1^) and nitrogen (N) (g kg^−1^) contents were determined using the Dumas method in a vario MACRO device (Elementar Analysensysteme, Hanau, Germany). Foliar phosphorus (P) (g kg^−1^) content was determined using an inductively coupled plasma-optical emission spectrometer (ICP-OES) (Varian 725ES, Varian Inc., Palo Alto, CA, USA).

### Respiration experiment

To determine soil and litter respiration, we conducted an *ex-situ* respiration experiment, using 96 experimental microcosms in an air-conditioned incubation room. For each of the 48 plots, we set up two soil environments by transferring 40 g air-dried soil to each of two air-tight glass jars (287 mL) fitted with two three-way valves to allow air sampling from the headspace. Soil was compacted to a bulk density of 1.5 g cm^−3^ and the water filled pore space was set to 60 wt% by adding demineralized water. The jars were incubated for 12 days at 25°C with open valves to allow free soil respiration. After this initial incubation, an amount of oven-dried, milled litter proportional to the litter available on the forest floor (tons ha^−1^; see above) was transferred to one of each set of two jars. Jars with only soil were used to determine heterotrophic soil respiration (R_S_; release of carbon from soil organic matter and from inorganic carbon sources from the soil by microorganisms). Jars with soil and litter were used to determine total respiration (R_LIT+S_; litter decomposition + soil respiration). Valves were closed and jars were placed at 25°C in a dark incubation room. R_S_ and R_LIT+S_ were determined by periodically measuring the CO_2_-concentration in the headspace of the jars by use of a LI-820 CO_2_ infrared gas analyzer (LI-COR Biosciences, Lincoln, Nebraska USA) attached in closed circuit to each jar separately. The gas in the closed circuit stream passes through a Mg(ClO_4_)_2_ (Sercon, UK) absorptive water trap to remove water vapor from the air sample. Between measurements, the circuit was flushed with CO_2_-free air by looping in a CO_2_ trap (Carbosorb, Sercon, UK). After each measurement, jar lids were removed for 5 min to allow CO_2_ concentrations to drop to background levels (see Kerré et al., 2015 for a similar protocol and see Supplementary Presentations 1 and 2 for the protocol and illustrations of the respiration experiment). A minimum of 12 and maximum of 16 measurements spread over a period of 25 days were taken for each jar. Each measurement represented the realized respiration over ~24 h.

Measured CO_2_ concentrations in ppm were corrected for atmospheric background concentrations (which were measured in three blank jars per batch) and then converted to instantaneous respiration rates (g CO_2_ ha^−1^ h^−1^) via the ideal gas law. Instantaneous respiration rates were converted to total CO_2_ released (kg ha^−1^) by integrating the instantaneous respiration rate curves over time. The slope of a linear regression fitted over the total CO_2_ released curve was then used as an estimator for the average respiration rate (kg CO_2_ ha^−1^ h^−1^) realized during the experiment in each jar. Because of expected (see e.g., Wang et al., 2016) and observed positive priming effects related to the sudden addition of biomass to the jars containing soil and litter, the first four measurements per jar were not taken into account when fitting the linear regressions.

### Statistical analysis

For all 48 plots, tree, and shrub GBH data were transformed to a measure of species dominance by converting GBH to basal area per hectare (BA; m^2^). The resulting data matrix was split into one vector describing the BA of *P. serotina* and one 25 × 48 matrix for the BA of the 25 indigenous woody species recorded during the field surveys. We used detrended correspondence analysis (DCA) to reduce the latter, multivariate data matrix to two DCA axes which reflect differences in species composition and may represent underlying environmental gradients. We used DCA because the structure in the dataset was too weak for non-metric multidimensional scaling (NMS). We used the DCA axes to represent differences in indigenous litter species composition and quality (via species and their traits; Makkonen et al., 2012), with DCA1 (41.3% variance explained; eigenvalue 0.873; gradient length 2.288) representing a turnover from broadleaved species to pines and DCA2 (16.4% variance explained; eigenvalue 0.330; gradient length 2.017) a turnover from *F. sylvatica* to *C. betulus*. Although these are rather short gradients, DCA has been found to produce robust results in similar cases (Ejrnaes, 2000). Similarly, we used principal component analysis (PCA) with a varimax rotation to reduce the soil chemical data matrix (soil pH, soil P, soil Ca, K, Mg, and Na content) to two PCA axes which reflect differences in soil properties, with PCA1 (45.2% variance explained) representing a gradient of soil pH (*r* = 0.900, *P* < 0.001) and base cations (soil Ca *r* = 0.973, *P* < 0.001; soil Mg *r* = 0.775, *P* < 0.001), and PCA2 (25.1% variance explained) a soil nutrient or NPK gradient (soil N *r* = 0.910, *P* < 0.001; soil P *r* = 0.734, *P* < 0.001; soil K *r* = 0.824, *P* < 0.001; soil Mg *r* = 0.384, *P* < 0.001).

The variables that have an effect on heterotrophic respiration are related to plant species composition and climate, and include soil temperature, soil moisture, amount, and quality of litter and soil organic matter, soil pH, soil nutrients and soil disturbance (e.g., Cornwell et al., 2008; DeForest et al., 2009; Wang et al., 2016). Under our controlled microcosm conditions, there was only minimal random variance in soil temperature, moisture, and disturbance between cases. We accounted for differences in soil properties between uninvaded and invaded stands to separate invasion effects from effects of soil chemical properties (existing or caused by *P. serotina*), as well as effects from litter quantity and quality. To that end, we built general linear models for the response variables R_S_ and R_LIT+S_ using invasion by *P. serotina* (0/1) as the fixed factor, and litter mass (tons ha^−1^), SOM content (wt%), DCA1 and DCA2 scores (measures for species composition, as proxy for litter species composition and litter quality; Cornwell et al., 2008), and PCA1 and PCA2 scores (for soil properties) as covariates. After evaluation of the initial full models, we built reduced general linear models for R_S_ and R_LIT+S_ using invasion by *P. serotina* (0/1) as the fixed factor, the significant (*P* < 0.05) covariates from the full models, and their interaction terms with the fixed *P. serotina* factor. The main effects of *P. serotina* on heterotrophic respiration rates were estimated by calculating estimated marginal means and 95% confidence intervals for R_S_ and R_LIT+S_ for stands invaded and not invaded by *P. serotina* based on these reduced general linear models.

To assess the effect of *P. serotina* invasion on canopy leaf chemical signatures, we built linear mixed models for foliar C, N, and P, and for foliar C:N and N:P ratios, for *C. betulus, F. sylvatica, Quercus* spp., and *P. sylvestris* using invasion by *P. serotina* (0/1) as fixed factor, and soil pH plus the relevant variables from the set SOM (wt%), soil N (wt%), and soil P (mg kg^−1^) as covariates. We used the plot ID as random grouping variable to account for replication of tree species within plots. The main effects of *P. serotina* on foliar nutrient contents and nutrient ratios were estimated by calculating estimated marginal means (i.e., controlling for the covariates) with 95% confidence intervals for foliar nutrient contents and ratios in *C. betulus, F. sylvatica, Quercus* spp., and *P. sylvestris* based on these linear mixed models. We specifically tested for significance in differences (i) between indigenous species and *P. serotina*; and (ii) within indigenous species between invaded and uninvaded stands.

Spatial analyses (calculating areas and distances between stands) were performed in QGIS 2.2.0. All statistical analyses were performed in IBM SPSS Statistics 20.

## Results

Soil chemical properties differed significantly between stands with and without *P. serotina* [Wilk's λ = 0.670; *F*_(8, 39)_ = 2.403; *P* = 0.033; Table 1]. Average soil pH and the concentrations of the basic cations K, Ca, and Mg were significantly lower in invaded stands (0.001 < *P* < 0.038; Table 1). Average SOM, soil N and the concentration of Na were also lower, and soil P was higher in invaded stands compared to uninvaded stands, but these differences were not statistically significant (0.103 < *P* < 0.455; Table 1).

**Table 1:** **Soil pH and soil nutrient concentrations in mixed deciduous forest stands uninvaded and invaded by the alien tree American black cherry (*Prunus serotina* Ehrh.) in the forest of Compiègne, France**.

	**95% confidence intervals for means**		***F***	***P***
	**Uninvaded (*N* = 20)**	**Invaded (*N* = 28)**		
pH	3.7–4.5	3.0–3.7	8.317	0.006^**^
SOM (mass%)	5.1–7.4	4.0–6.0	2.770	0.103
N (mass%)	0.14–0.20	0.13–0.18	0.567	0.455
P (mg kg^−1^)	9.9–18.5	13.9–20.4	0.824	0.369
K (cmol kg^−1^)	0.13–0.17	0.10–0.14	4.553	0.038^*^
Ca (cmol kg^−1^)	4.46–8.10	0.70–3.78	11.621	0.001^**^
Mg (cmol kg^−1^)	0.26–0.42	0.14–0.27	6.525	0.014^*^
Na (cmol kg^−1^)	0.30–0.35	0.28–0.32	2.969	0.092

*Stands were considered invaded when the basal area of P. serotina exceeded 0.05 m^2^ ha^−1^. F-tests for between subjects effects of P. serotina presence are based on a multivariate general linear model. Overall effect of fixed factor P. serotina presence: Wilks' lambda = 0.670, F_(8, 39)_ = 2.403, P = 0.033*.

Significant differences between invaded and uninvaded stands are indicated by

* P < 0.05 and

** *P < 0.01*.

**Figure 1:**
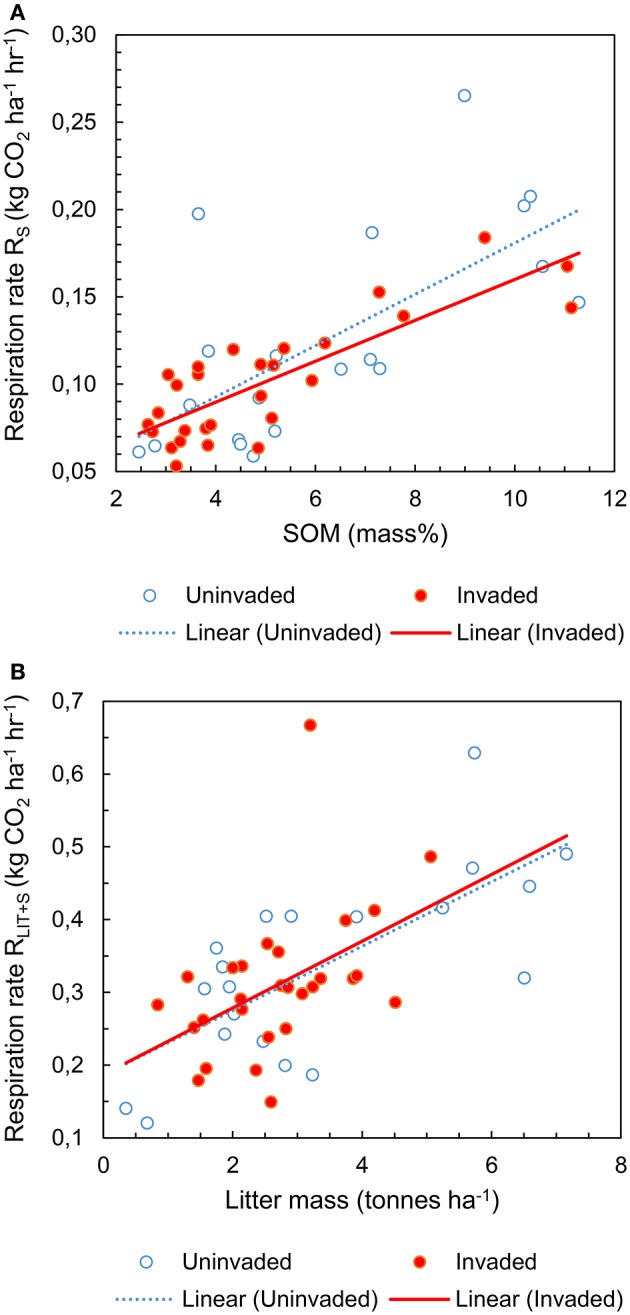
**Heterotrophic respiration rates of soil (R_S_) as a function of soil organic matter content (A)**; and of soil and litter combined (R_LIT+S_) as a function of litter availability on the forest floor **(B)**, determined for mixed deciduous forest stands uninvaded (open symbols, *N* = 20) and invaded (full symbols, *N* = 28, basal area of *P. serotina* > 0.05 m^2^/ha) by the alien invasive American black cherry (*Prunus serotina* Ehrh.) in the forest of Compiègne, France.

**Figure 2:**
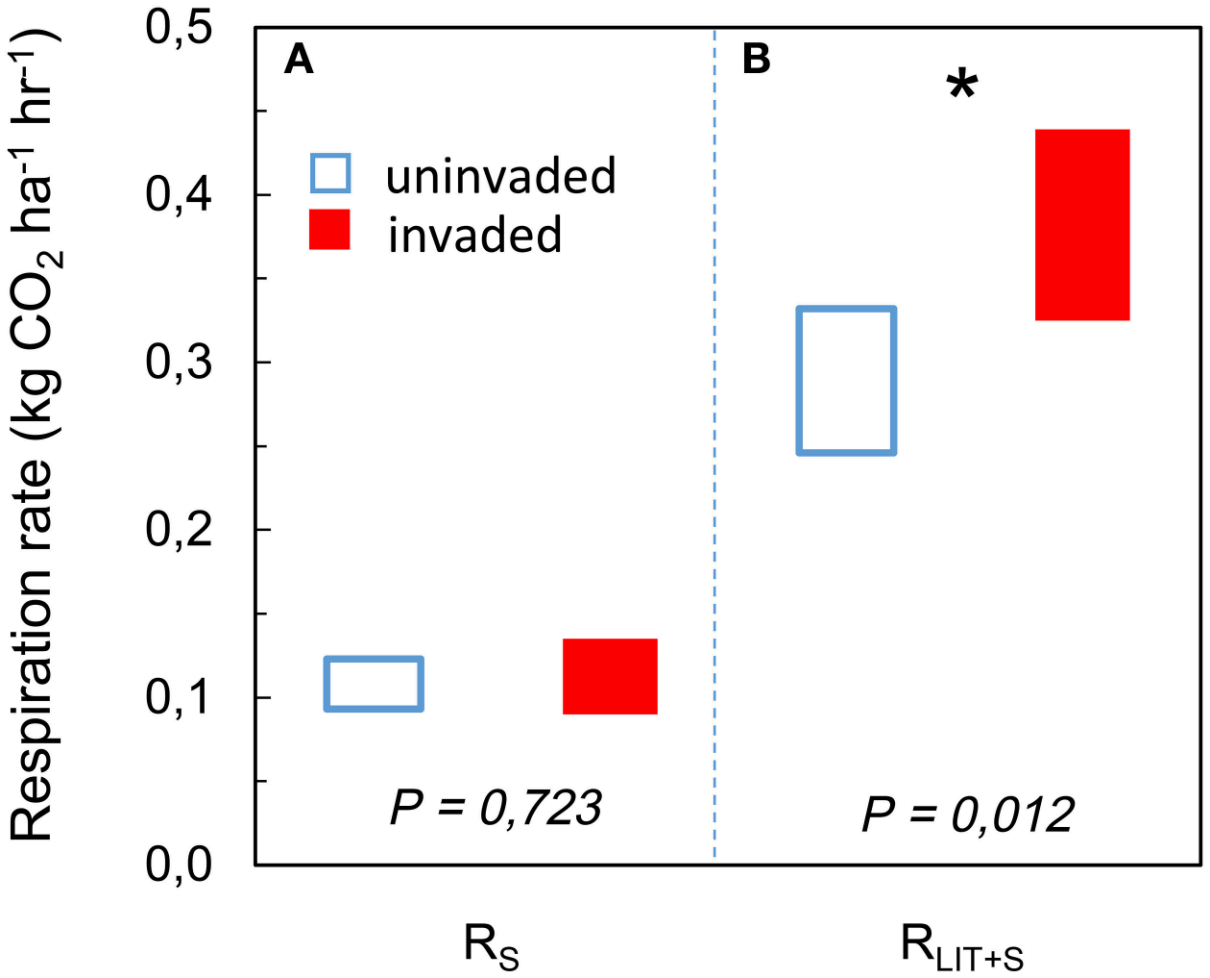
**Estimated marginal mean heterotrophic respiration rates (95% CI) of soil (R_S_) after accounting for variability in soil pH and soil organic matter (SOM) content (A)**; and of soil and litter combined (R_LIT+S_) after accounting for variability in soil pH and litter availability **(B)**, determined for mixed deciduous forest stands uninvaded (open bars, *N* = 20) and invaded (full bars, *N* = 28, basal area of *P. serotina* > 0.05 m^2^/ha) by the alien invasive American black cherry (*Prunus serotina* Ehrh.) in the forest of Compiègne, France. Covariates appearing in the models were evaluated at soil PCA1 = 0 and **(A)** SOM = 5.5 mass% and **(B)** litter mass = 2.97 tons ha^−1^. The total heterotrophic respiration rate R_LIT+S_ was significantly higher (^*^), i.e., by 32%, in stands invaded by *P. serotina*.

The respiration rate from soil R_S_ increased with available SOM [*F*_(1, 42)_ = 47.267; *P* < 0.001; Figure 1A] but there was no significant effect of *P. serotina* [*F*_(1, 42)_ = 0.199; *P* = 0.658; Table S1]. The respiration rate from soil and litter combined R_LIT+S_ increased with available litter on the forest floor [*F*_(1, 42)_ = 23.562; *P* < 0.001; Figure 1B] and with increasing soil pH and concentrations of base cations Ca and Mg [soil PCA1: *F*_(1, 42)_ = 9.320; *P* = 0.004]; there was a significant effect of *P. serotina* on the combined respiration rate from soil and litter [*F*_(1, 42)_ = 5.351; *P* = 0.026; Table S2]. Accounting for the significant random effects, the average heterotrophic respiration from soil R_S_ was 5.6% higher in stands invaded by *P. serotina*, but not significantly different from uninvaded stands [*F*_(1, 42)_ = 0.127; *P* = 0.723; Figure 2; estimated marginal means ± SE, uninvaded 0.108 ± 0.007 vs. invaded: 0.114 ± 0.006 kg CO_2_ ha^−1^ h^−1^]. The average heterotrophic respiration from soil and litter R_LIT+S_ was significantly higher, by 32%, in stands invaded by *P. serotina* than in non-invaded stands [*F*_(1, 42)_ = 6.816; *P* = 0.012; Figure 2; estimated marginal means ± SE, uninvaded 0.289 ± 0.021 vs. invaded: 0.382 ± 0.028 kg CO_2_ ha^−1^ h^−1^].

**Figure 3:**
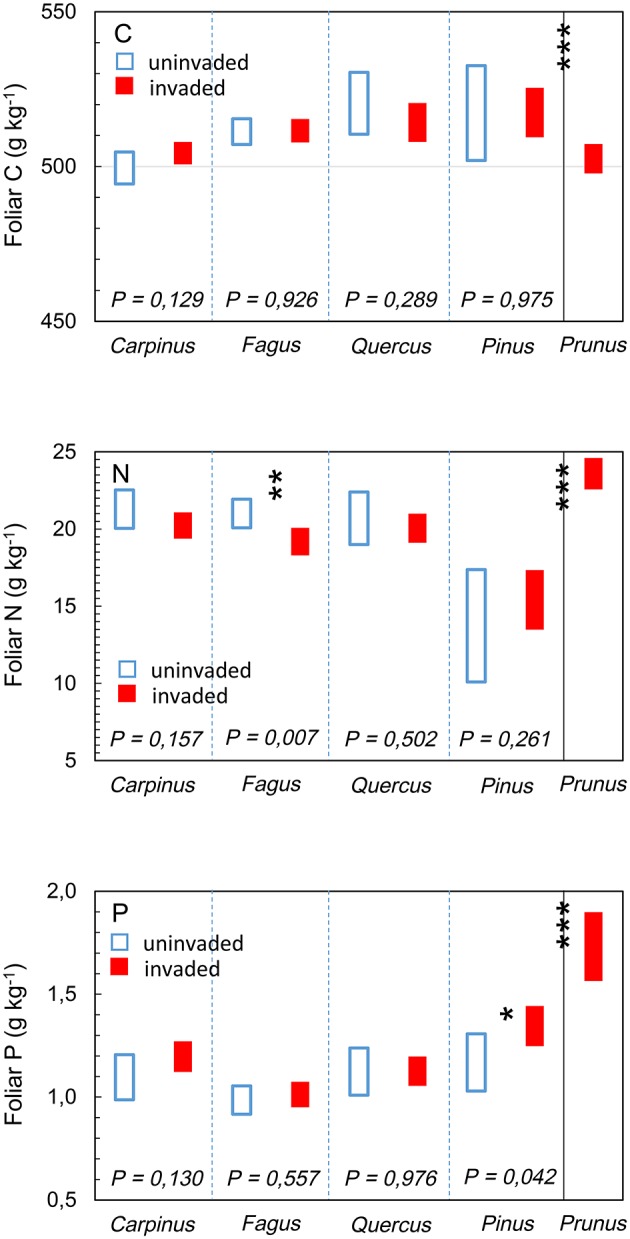
**Estimated marginal mean (95% CI) foliar carbon (C) content, after accounting for variability in soil pH and soil organic matter content; foliar nitrogen (N) content, after accounting for variability in soil pH and soil N content; and foliar phosphorous (P) content, after accounting for variability in soil pH and soil P content of upper canopy light leaves in mixed deciduous forest stands uninvaded (open bars, *N* = 96 trees in 20 plots) and invaded (full bars, *N* = 147 trees in 28 plots, basal area of *P. serotina* > 0.05 m^2^/ha) by the alien invasive American black cherry *Prunus serotina* (right panel, *N* = 64 trees) in the forest of Compiègne, France**. Means are for hornbeam *Carpinus betulus* (uninvaded 24 trees vs. invaded 36 trees), beech *Fagus sylvatica* (uninvaded 45 trees vs. invaded 42 trees), oaks *Quercus* spp. (*Q. robur* and *Q. petraea*; uninvaded 21 trees vs. invaded 57 trees), Scots pine *Pinus sylvestris* (uninvaded 12 samples from 6 trees vs. invaded 24 samples from 12 trees) and American black cherry *Prunus serotina* (64 trees). Differences between *Prunus serotina* and indigenous species as a group were significant (^***^all *P* < 0.001); significant differences between invaded and uninvaded stands are indicated by ^*^P < 0.05 and ^**^P < 0.01 (type III *F*-test of fixed effect of *P. serotina*).

**Figure 4:**
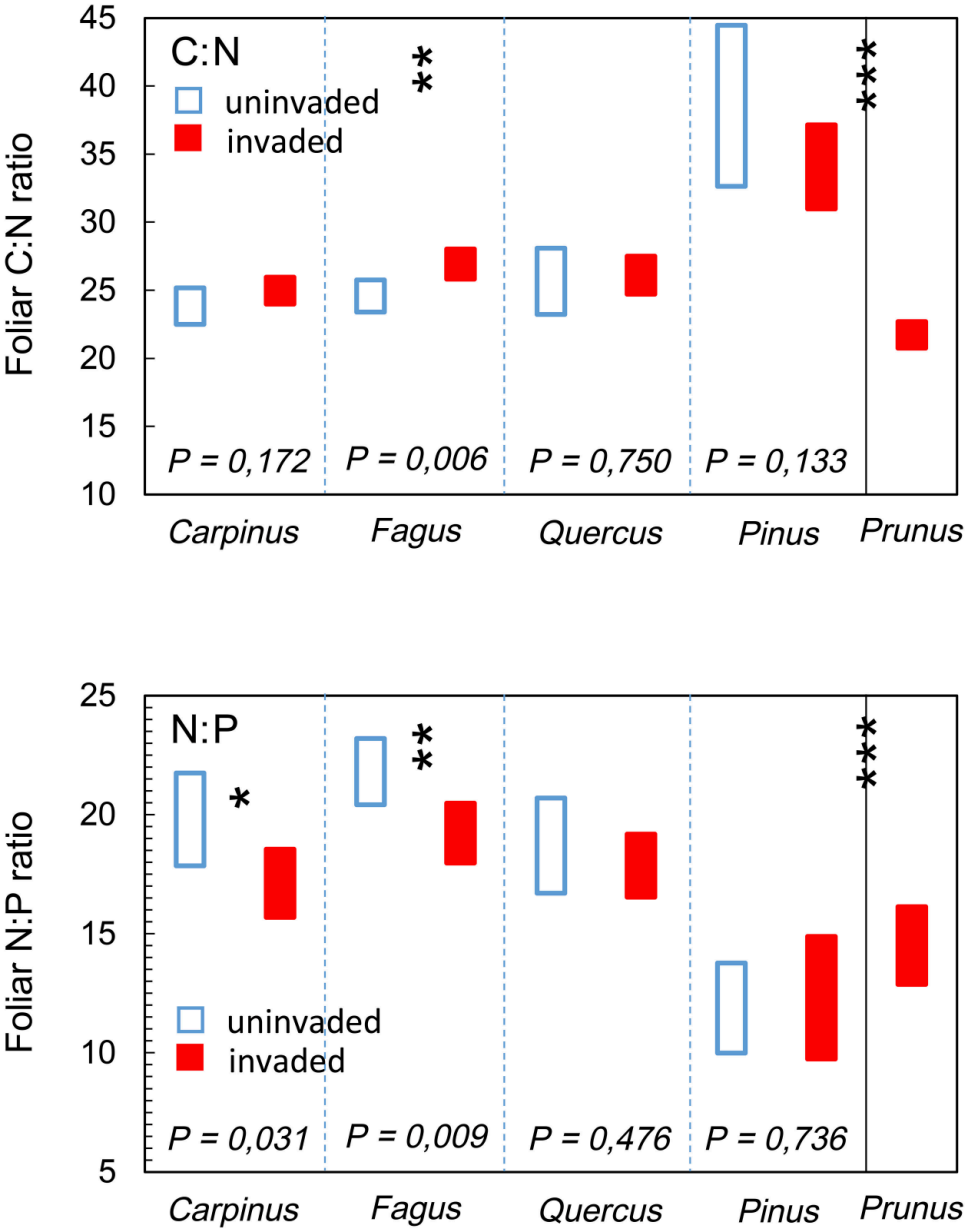
**Estimated marginal mean (95% CI) foliar carbon-nitrogen ratio (C:N), after accounting for variability in soil pH, soil organic matter content and soil N content; and foliar nitrogen-phosphorous ratio (N:P), after accounting for variability in soil pH, soil N, and soil P content of upper canopy light leaves in mixed deciduous forest stands uninvaded (open bars, *N* = 96 trees in 20 plots) and invaded (full bars, *N* = 147 trees in 28 plots, basal area of *P. serotina* > 0.05 m^2^/ha) by the alien invasive American black cherry (*Prunus serotina* Ehrh.; right panel, *N* = 64 trees) in the forest of Compiègne, France**. Means are for hornbeam *Carpinus betulus* (uninvaded 24 trees vs. invaded 36 trees), beech *Fagus sylvatica* (uninvaded 45 trees vs. invaded 42 trees), oaks *Quercus* spp. (*Q. robur* and *Q. petraea*; uninvaded 21 trees vs. invaded 57 trees), Scots pine *Pinus sylvestris* (uninvaded 12 samples from 6 trees vs. invaded 24 samples from 12 trees) and American black cherry *Prunus serotina* (64 trees). Differences between *P. serotina* and indigenous species as a group were significant (^***^ all *P* < 0.001); significant differences between invaded and uninvaded stands are indicated by ^*^P < 0.05 and ^**^P < 0.01 (type III *F*-test of fixed effect of *P. serotina*).

The foliar chemical signature of *P. serotina* was significantly different from that of the indigenous species combined [foliar C: *F*_(1, 42)_ = 19.562; foliar N: *F*_(1, 316)_ = 154.161; foliar P: *F*_(1, 324)_ = 235.652; all *P* < 0.001; Figure 3]. Consequently, also foliar nutrient ratios C:N and N:P of *P. serotina* were significantly different from the indigenous species [foliar C:N *F*_(1, 313)_ = 131.226; foliar N:P *F*_(1, 304)_ = 16.319; both *P* < 0.001; Figure 4]. Accounting for variability in soil chemical properties (soil pH, SOM, soil N, and soil P), the average foliar C content of *P. serotina* was 1.6% lower, and the average foliar N and foliar P content higher, respectively by 24.0 and 50.7 %, than the averages observed in the indigenous trees (all *P* < 0.001; Figure 3). The C:N and N:P ratios of *P. serotina* leaves were, respectively, 22.4 and 10.1% lower than the averages observed in the indigenous trees (both *P* < 0.001; Figure 4).

The effects of *P. serotina* presence on the foliar chemical properties of the indigenous trees differed between species. There were no significant differences in leaf C content, which were generally within the range of the global average of 500 g kg^−1^ (Figure 3, panel C; all *P* > 0.05). Accounting for variability in soil pH and soil N, the foliar N content of *F. sylvatica* was 8.7 % lower [*F*_(1, 229)_ = 8.492; *P* = 0.007; Figure 3, panel N] in stands invaded by *P. serotina* (estimated marginal means ± SE, uninvaded 21.0 ± 0.5 vs. invaded: 19.2 ± 0.4 g kg^−1^). Average foliar N content was also lower in *C. betulus* and *Quercus* spp., and higher in *P. sylvestris* in stands invaded by *P. serotina* but these differences were not significant (*P* = 0.157, 0.502, and 0.261, respectively; Figure 3, panel N).

Accounting for variability in soil pH and soil P, the foliar P content of *P. sylvestris* was 12.3% higher [*F*_(1, 34)_ = 4.462; *P* = 0.042; Figure 3] in stands invaded by *P. serotina* (estimated marginal means ± SE, uninvaded 1.17 ± 0.07 vs. invaded: 1.35 ± 0.05 g kg^−1^). Foliar P contents of the broadleaved species did not differ significantly between invaded and uninvaded stands (0.130 < *P* <0.976; Figure 3, panel P).

Differences in foliar N and P resulted in significant differences in foliar nutrient ratios for *F. sylvatica* and *C. betulus*. Accounting for variability in soil pH and soil C and soil N contents, the foliar C:N ratio of *F. sylvatica* was 9.5 % higher [*F*_(1, 30)_ = 8.754; *P* = 0.006; Figure 4, panel C:N] in stands invaded by *P. serotina* (estimated marginal means ± SE, uninvaded 24.6 ± 0.6 vs. invaded: 26.9 ± 0.5). *P. serotina* had a positive (but not significant, *P* = 0.133) effect on the C:N ratio in *P. sylvestris* (estimated marginal means ± SE, uninvaded 38.6 ± 2.0 vs. invaded: 34.1 ± 1.2). Accounting for variability in soil pH and soil N and soil P contents, the foliar N:P ratios of *F. sylvatica* and *C. betulus* were 11.8 and 13.5% lower, respectively [*F. sylvatica*: *F*_(1, 28)_ = 8.000; *P* = 0.009; *C. betulus*: *F*_(1, 19)_ = 5.372; *P* = 0.032; Figure 4, panel N:P] in stands invaded by *P. serotina* (*F. sylvatica*: estimated marginal means ± SE, uninvaded 21.8 ± 0.7 vs. invaded: 19.2 ± 0.6; *C. betulus*: uninvaded 19.8 ± 0.9 vs. invaded: 17.1 ± 0.7).

## Discussion

We demonstrated that there are differences in soil chemical properties between invaded and uninvaded stands. Our results also showed that *P. serotina* has a number of effects that alter the ecological processes of the forest ecosystem, by affecting relative nutrient contents in overstory light leaves and by impacting carbon dynamics in the pedosphere. More specific, our results suggest that (1) *P. serotina* possesses higher foliar N and P and lower C:N and N:P ratios compared to the indigenous species, which indicates high litter quality; (2) pine trees have higher foliar P and C:N in the presence of *P. serotina*; and (3) *F. sylvatica* and *C. betulus* have lower foliar N:P ratios in the presence of *P. serotina*. If differences in fresh leaf chemistry translate to similar differences in litter chemistry, *P. serotina* could change ecosystem nutrient cycling by improving overall litter quality, not only by adding its own nutrient-rich litter, but also by improving the litter quality of pines and through subtle changes in relative proportions of nutrients in broadleaved species. Conversely, our results also suggest that the presence of *P. serotina* is associated with higher heterotrophic respiration rates that suggest increased proportions of labile litter, and that *P. serotina* presence is negatively associated with foliar N and C:N ratio in indigenous broadleaved species, in particular *F. sylvatica*.

### Higher soil acidity and lower base cation concentrations in invaded stands

Soils in invaded stands were characterized by significantly lower soil pH and lower concentrations of base cations (Table 1). The literature on *P. serotina* effects on forest soils is ambiguous. Some studies report decreases in soil acidity and increases in concentrations of soil nutrients through the perceived positive litter effect of *P. serotina* (Vanderhoeven et al., 2005; Dassonville et al., 2008). Other studies indicate that the high biomass production of *P. serotina* requires an increased uptake of soil nitrogen and base cations, resulting in increased soil acidity (Starfinger et al., 2003). Thus, depending on the relative contributions of the positive litter effect and the negative N and cation depletion effects, invasions of *P. serotina* may yield decreases or increases in soil acidity (and thus improve or degrade soil quality). It is important to note that *P. serotina* has been shown to possess a competitive advantage on poor, sandy soils, and its wide distribution in pine forests throughout Europe has been attributed to it (Lorenz et al., 2004). Thus, the higher soil acidity in invaded stands may at least partially reflect the initial high soil acidity of the stands prior to invasion, which therefore may have been easier to invade. The resulting invasion by *P. serotina* may have caused a positive feedback, further increasing soil acidity and decreasing soil cation concentration through rapid growth. These results underscore that it is important to adequately account for environmental variability when assessing *P. serotina* effects on the ecosystem.

### *Prunus serotina* short-circuits nutrient fluxes

*P. serotina* had higher foliar N and P concentrations than the studied indigenous species (Figure 3). The leaf carbon concentration and the C:N ratio of *P. serotina* were higher than those observed in its native range (Heberling et al., 2016). Such differences may stem from the ability of invasive species to adapt their resource use strategy in the invasive range (Heberling et al., 2016) and hereby gaining the ability to use the available resources more efficiently than the co-occurring native species (e.g., Baruch and Goldstein, 1999; Rothstein et al., 2004). Some evidence indeed suggests that *P. serotina* taps from a larger nutrient pool than the indigenous species on the same site (Dassonville et al., 2008). The resulting high foliar N and P may be responsible for the fast decomposition of *P. serotina* litter and such leaf properties may improve overall litter dynamics and humus formation (Lorenz et al., 2004) as well as accelerate nutrient circulation at the level of the ecosystem (Carreño-Rocabado et al., 2012; Aragón et al., 2014). In contrast, the lower foliar N content observed in the indigenous tree species (Figure 3), and in particular *F. sylvatica*, provides support for the hypothesis that the invasive *P. serotina* is more efficient in exploiting soil N resources than its indigenous competitors (Vilà and Weiner, 2004; Dassonville et al., 2008), and may effectively short-circuit the forest's nutrient cycles in response to its high N demand (Lorenz et al., 2004). This may have important consequences for the ecosystem in terms of humus quality, as the dominant species *F. sylvatica* and *Quercus* spp. are species with an intrinsic relatively poor chemical litter quality (compared to other, indigenous deciduous species such as *C. betulus* and *Tilia cordata*; Jacob et al., 2010). A decrease in their foliar N, and thus increase in foliar C:N ratio (Figure 4), further deteriorates the quality of the beech and oak litter, and this may result in ecosystem degradation through soil acidification (contributing to the above mentioned positive feedback loop) and impacts on humification. The degradation of the overstory litter quality may therefore partially offset the effects of the nutrient-rich and fast decomposing *P. serotina* litter (Lorenz et al., 2004; Ashton et al., 2005). This may also explain why dense, nutrient-rich shrub layers with high litter quality, including litter from *P. serotina*, could not mitigate soil acidification in pine and oak forests on poor sandy soils in NE Belgium (Van Nevel et al., 2014).

An important long-term effect of reduced foliar N contents in the presence of *P. serotina* may have consequences that exceed the ecosystem boundaries. As foliar N content is related to the leaf light-saturated rate of photosynthesis and the leaf respiration rate and thus an important driver of photosynthetic capacity (Wright et al., 2004; Hikosaka et al., 2016), reductions in foliar N content in the long-lived indigenous trees may reduce their net primary productivity, and thus carbon sequestration capacity. Because net primary production is usually higher in invaded ecosystems (Liao et al., 2007; Vilà et al., 2011), potential losses in carbon storage in indigenous tree species following invasion would likely be compensated by the fast-growing *P. serotina*, either in the soil or in its biomass. Carbon storage in stem wood accounts for 80% of net carbon sequestration by forests in Europe (de Vries et al., 2006), and here *P. serotina* may be less efficient than the indigenous species in the long term. *P. serotina* is relatively short-lived compared to the indigenous broadleaved species, and its wood is currently not used in forest products with long life cycles. Therefore, it is unlikely that *P. serotina* can serve as a long term carbon store as efficiently as the oaks and beech trees could do.

### Accelerated carbon turnover in invaded stands

Biotic invasions alter and often reduce the functional diversity of ecosystems (Chabrerie et al., 2010) and reductions of litter types, in turn, have been linked to slower litter carbon cycling (Handa et al., 2014). In our experiment, heterotrophic respiration was significantly higher in stands invaded by *P. serotina* when comparing respiration from soil and litter combined (R_LIT+S_), but not when comparing respiration from soil only. In an *in vitro* carbon mineralization experiment, Koutika et al. (2007) also found little evidence for an effect of *P. serotina* on carbon mineralization from soil, and therefore, our observed *P. serotina* effect on respiration may primarily be mediated by litter (see also DeForest et al., 2009). In annual grasslands, it has been demonstrated that rapidly decomposing litter from an exotic grass accelerated the decomposition of native litter in litter mixtures, hereby enhancing soil respiration rates and accelerating carbon cycling (Zhang et al., 2014), rather than slowing down litter carbon cycling through effects on community functional diversity. In the same grassland ecosystem, exotic forbs also increased soil respiration via their high amounts of rapidly decomposing litter (Zhang et al., 2016).

Increased respiration in the presence of litter does not imply that our observed additional respiration R_+_ (calculated as R_LIT+S_ − R_S_) can be entirely allocated to litter and thus equals R_LIT_. Nutrients present in litter biomass have an effect on the soil microbial communities and their activity (Wang et al., 2016). Because of a positive priming effect of these litter nutrients and because of synergistic effects observed elsewhere in mixtures of native (Handa et al., 2014) and of native and exotic litter (Rothstein et al., 2004; Hickman et al., 2013; Zhang et al., 2014), the respiration from soil may be higher in the presence of litter (DeForest et al., 2009), and in particular in the presence of large quantities of exotic *P. serotina* litter. *In situ* measurements would increase our understanding of the effect of *P. serotina* on carbon turnover, as soil microclimate, roots and microbial communities also have an influence on respiration rates. But as our measured *ex situ* soil and litter incubation is a good indicator of SOM and litter quality, we can conclude that invasion by *P. serotina* in any case increases the proportion of labile litter in the forest.

## Conclusions

Our analyses suggest that *P. serotina* does not always improve soil and overall litter quality despite its high quality litter and positive effect on pine foliar chemistry. It is possible that *P. serotina* is changing nitrogen, phosphorus and carbon cycles to its own advantage, hereby increasing carbon turnover via labile litter, affecting the relative nutrient contents in the overstory leaves, and potentially altering the photosynthetic capacity of the long-lived indigenous broadleaved keystone species. Our results support the classification of *P. serotina* as an invasive species with a negative impact on its environment, but more studies are needed to confirm the ecosystem engineering role of this widespread invasive species. This is important because uncontrolled invasion of European temperate forests by *P. serotina* may affect the climate change mitigation potential of these forests in the long term, through additive effects on local nutrient cycles.

## Ethics statement

To comply with Belgian and EU firearms legislation, RA obtained a Belgian weapon license Model 4 (Nr. 4/200014/15/15013010) to operate a firearm for scientific purposes and a European Firearms Pass (Nr. 20/21/15/14045), both from the Office of the Governor of the Province of Flemish Brabant.

## Author contributions

BS, RV, JL, HF, SSc, GD, and OH conceived the study. RA, ME, SSk, and JL established plots, collected soil, and litter samples and conducted field and laboratory measurements. RA carried out soil carbon analyses, performed the respiration experiment and, together with ME, MN, JP, and JL, sampled canopy leaves by shotgun. SSk coordinated soil chemical analysis. JL coordinated foliar chemical analysis. RA performed data analysis, wrote the initial manuscript with OH and revised the manuscript. All authors contributed to the interpretation of the results and read and approved the final manuscript.

## Funding

This study was supported by the ERA-Net BiodivERsA, with the national funders Agence Nationale de la Recherche (ANR), the Belgian Science Policy Office (BELSPO), and the German Research Foundation (DFG).

### Conflict of interest statement

The authors declare that the research was conducted in the absence of any commercial or financial relationships that could be construed as a potential conflict of interest.

## References

[B1] Aragón R. Montti L. Ayup M. M. Fernández R. (2014). Exotic species as modifiers of ecosystem processes: litter decomposition in native and invaded secondary forests of NW Argentina. Acta Oecol. 54, 21–28. 10.1016/j.actao.2013.03.007

[B2] Ashton I. W. Hyatt L. A. Howe K. M. Gurevitch J. Lerdau M. T. (2005). Invasive species accelerate decomposition and litter nitrogen loss in a mixed deciduous forest. Ecol. Appl. 15, 1263–1272. 10.1890/04-0741

[B3] Baruch Z. Goldstein G. (1999). Leaf construction cost, nutrient concentration, and net CO_2_ assimilation of native and invasive species in Hawaii. Oecologia 1221, 183–192. 10.1007/s004420050920 28308558

[B4] Belnap J. Phillips S. L. Sherrod S. K. Moldenke A. (2005). Soil biota can change after exotic plant invasion: does this affect ecosystem processes? Ecology 86, 3007–3017. 10.1890/05-0333

[B5] Buysse W. (2012). Realizing Management Goals in the Presence of Black Cherry (in Dutch). Brussels: Agentschap voor Natuur en Bos.

[B6] Carreño-Rocabado G. Peña-Claros M. Bongers F. Alarcón A. Licona J. C. Poorter L. (2012). Effects of disturbance intensity on species and functional diversity in a tropical forest. J. Ecol. 100, 1453–1463. 10.1111/j.1365-2745.2012.02015.x

[B7] Chabrerie O. Loinard J. Perrin S. Saguez R. Decocq G. (2010). Impact of *Prunus serotina* invasion on understory functional diversity in a European temperate forest. Biol. Invasions 12, 1891–1907. 10.1007/s10530-009-9599-9

[B8] Chabrerie O. Roulier F. Hoeblich H. Sebert-Cuvillier E. Closset-Kopp D. Leblanc I.. (2007). Defining patch mosaic functional types to predict invasion patterns in a forest landscape. Ecol. Appl. 17, 464–481. 10.1890/06-0614 17489253

[B9] Chabrerie O. Verheyen K. Saguez R. Decocq G. (2008). Disentangling relationships between habitat conditions, disturbance history, plant diversity and American black cherry (*Prunus serotina* Ehrh.) invasion in a European temperate forest. Divers. Distrib. 14, 204–212. 10.1111/j.1472-4642.2007.00453.x

[B10] Chen Y. Chen G. Ye Y. (2015). Coastal vegetation invasion increases greenhouse gas emission from wetland soils but also incrases soil carbon accumulation. Sci. Tot. Environ. 526, 19–28. 10.1016/j.scitotenv.2015.04.077 25918889

[B11] Chytrý M. Wild J. Pyšek P. Jarošík V. Dendoncker N. Reginster I. (2011). Projecting trends in plant invasions in Europe under different scenarios of future land-use change. Glob. Ecol. Biogeogr. 21, 75–87. 10.1111/j.1466-8238.2010.00573.x

[B12] Closset-Kopp D. Chabrerie O. Valentin B. Delachapelle H. Decocq G. (2007). When Oskar meets alice: does a lack of trade-off in *r/K*-strategies makes *Prunus serotina*. a successful invader of European forests? Forest Ecol. Manage. 247, 120–130. 10.1016/j.foreco.2007.04.023

[B13] Cornwell W. K. Cornelissen J. H. C. Amatangelo K. Dorrepaal E. Eviner V. T. Godoy O.. (2008). Plant species traits are the predominant control on litter decomposition rates within biomes worldwide. Ecol. Lett. 11, 1065–1071. 10.1111/j.1461-0248.2008.01219.x 18627410

[B14] Dassonville N. Vanderhoeven S. Vanparys V. Hayez M. Gruber W. Meerts P. (2008). Impacts of alien invasive plants on soil nutrients are correlated with initial site conditions in NW Europe. Oecologia 157, 131–140. 10.1007/s00442-008-1054-6 18491146

[B15] de Vries W. Reinds G. J. Gundersen P. Sterba H. (2006). The impact of nitrogen deposition on carbon sequestration in European forests and forest soils. Global Change Biol. 12, 1151–1173. 10.1111/j.1365-2486.2006.01151.x

[B16] DeForest J. L. Chen J. McNulty S. G. (2009). Leaf litter is an important mediator of soil respiration in an oak-dominated forest. Int. J. Biometeorol. 53, 127–134. 10.1007/s00484-008-0195-y 19037664

[B17] Ehrenfeld J. G. (2003). Effects of exotic plant invasions on soil nutrient cycling processes. Ecosystems 6, 503–523. 10.1007/s10021-002-0151-3

[B18] Ejrnaes R. (2000). Can we trust gradients extracted by detrended correspondence analysis? J. Veget. Sci. 11, 565–572. 10.2307/3246586

[B19] Godefroid S. Phartyal S. S. Weyembergh G. Koedam N. (2005). Ecological factors controlling the abundance of non-native invasive black cherry (*Prunus serotina*) in deciduous forest understory in Belgium. Forest Ecol. Manage. 210, 91–105. 10.1016/j.foreco.2005.02.024

[B20] Halarewicz A. Żołnierz L. (2014). Changes in the understory of mixed coniferous forest plant communities dominated by the American black cherry (*Prunus serotina* Ehrh.). Forest Ecol. Manage. 313, 91–97. 10.1016/j.foreco.2013.11.006

[B21] Handa T. I Aerts R. Berendse F. Berg M. P. Bruder A. Butenschoen O.. (2014). Consequences of biodiversity loss for litter decomposition across biomes. Nature 509, 218–221. 10.1038/nature13247 24805346

[B22] Heberling J. M. Kichey T. Decocq G. Fridley J. D. (2016). Plant functional shifts in the invaded range: a test with reciprocal forest invaders of Europe and North America. Funct. Ecol. 30, 875–884. 10.1111/1365-2435.12590

[B23] Hejda M. Pyšek P. Jarošik V. (2009). Impact of invasive plants on the species richness, diversity and composition of invaded communities. J. Ecol. 97, 393–403. 10.1111/j.1365-2745.2009.01480.x

[B24] Hickman J. E. Ashton I. W. Howe K. M. Lerdau M. T. (2013). The native-invasive balance: implications for nutrient cycling in ecosystems. Oecologia 173, 319–328. 10.1007/s00442-013-2607-x 23443354

[B25] Hikosaka K. Noguchi K. Terashima I. (2016). Modeling leaf gas exchange, in Canopy Photosynthesis: from Basics to Applications, eds Hikosaka K. Niinemets Ü. Anten N. P. R. (New-York, NY: Springer-Verlag), 61–100.

[B26] Hoogsteen M. J. J. Lantinga E. A. Bakker E. J. Groot J. C. J. Tittonell P. A. (2015). Estimating soil organic carbon through loss on ignition: effects of ignition conditions and structural water loss. Eur. J. Soil Sci. 66, 320–328. 10.1111/ejss.12224

[B27] Hulme P. E. Pyšek P. Nentwig W. Vilà M. (2009). Will threat of biological invasions unite the European Union? Science 324, 40–41. 10.1126/science.1171111 19342572

[B28] Jacob M. Viedenz K. Polle A. Thomas F. M. (2010). Leaf litter decomposition in temperate deciduous forest stands with a decreasing fraction of beech (*Fagus sylvatica*). Oecologia 164, 1083–1094. 10.1007/s00442-010-1699-9 20596729 PMC2981742

[B29] Kerré B. Hernandez-Soriano M. C. Smolders E. (2015). Partitioning of carbon sources among functional pools to investigate short-term priming effects of biochar in soil: A ^13^C study. Sci. Tot. Environ. 547, 30–38. 10.1016/j.scitotenv.2015.12.107 26780129

[B30] Klotz S. (2007). Prunus serotina Fact Sheet. Delivering Alien Invasive Species Inventories for Europe (DAISIE). Available online at: http://www.europe-aliens.org/pdf/Prunus_serotina.pdf

[B31] Koutika L. S. Vanderhoeven S. Chapuis-Lardy L. Dassonville N. Meerts P. (2007). Assessment of changes in soil organic matter after invasion by exotic plant species. Biol. Fertil. Soils 44, 331–341. 10.1007/s00374-007-0210-1

[B32] Kramer T. D. Warren R. J. I. I Tang Y. Bradford M. A. (2012). Grass invasions across a regional gradient are associated with declines in belowground carbon pools. Ecosystems 15, 1271–1282. 10.1007/s10021-012-9583-6

[B33] Lazzaro L. Giuliani C. Fabiani A. Agnelli A. A. Pastorelli R. Lagomarsino A.. (2014). Soil and plant changing after invasion: the case of *Acacia dealbata* in a Mediterranean ecosystem. Sci. Tot. Environ. 497–498, 491–498. 10.1016/j.scitotenv.2014.08.014 25151267

[B34] Liao C. Peng R. Luo Y. Zhou X. Wu X. Fang C.. (2007). Altered ecosystem carbon and nitrogen cycles by plant invasion: a meta-analysis. New Phytol. 177, 706–714. 10.1111/j.1469-8137.2007.02290.x 18042198

[B35] Lorenz K. Preston C. M. Krumrei S. Feger K. H. (2004). Decomposition of needle/leaf litter from Scots pine, black cherry, common oak and European beech at a conurbation forest site. Eur. J. Forest. Res. 123, 177–188. 10.1007/s10342-004-0025-7

[B36] Makkonen M. Berg M. P. Handa I. T. Hättenschwiler S. van Ruijven J. van Bodegom P. M.. (2012). Highly consistent effects of plant litter identity and functional traits on decomposition across a latitudinal gradient. Ecol. Lett. 15, 1033–1041. 10.1111/j.1461-0248.2012.01826.x 22732002

[B37] Reinhart K. Rinella M. J. (2016). A common soil handling technique can generate incorrect estimates of soil biota effects on plants. New Phytol. 210, 786–789. 10.1111/nph.13822 26738893

[B38] Richter R. Berger U. E. Dullinger S. Essl F. Leitner M. Smith M. (2013). Spread of invasive ragweed: climate change, management and how to reduce allergy cost. J. Appl. Ecol. 50, 1422–1430. 10.1111/1365-2664.12156

[B39] Rothstein D. E. Vitousek P. M. Simmons B. L. (2004). An exotic tree alters decomposition and nutrient cycling in a Hawaiian montane forest. Ecosystems 7, 805–814. 10.1007/s10021-004-0009-y

[B40] Sitzia T. Campagnaro T. Kowarik I. Trentanovi G. (2016). Using forest management to control invasive alien species: helping implement the new European regulation on invasive alien species. Biol. Invasions 18, 1–7. 10.1007/s10530-015-0999-8

[B41] Starfinger U. Kowarik I. Rode M. Schepker H. (2003). From desirable ornamental plant to pest to accepted addition to the flora? – the perception of an alien tree species through the centuries. Biol. Invasion 5, 323–335. 10.1023/B:BINV.0000005573.14800.07

[B42] Thijs K. W. Brys R. Verboven H. A. F. Hermy M. (2012). The influence of an invasive plant species on the pollination success and reproductive output of three riparian plant species. Biol. Invasions 14, 355–365. 10.1007/s10530-011-0067-y

[B43] Urban J. Tatarinov F. Nadezhdina N. Cermák J. Ceulemans R. (2009). Crown structure and leaf aera of the understory species *Prunus serotina*. Trees Struct. Funct. 23, 391–399. 10.1007/s00468-008-0288-6

[B44] Van Nevel L. Mertens J. De Schrijver A. De Neve S. Verheyen K. (2014). Can shrub species with higher litter quality mitigate soil acidification in pine and oak forests on poor sandy soils? Forest Ecol. Manage. 330, 38–45. 10.1016/j.foreco.2014.07.002

[B45] Vanderhoeven S. Dassonville N. Meerts P. (2005). Increased topsoil mineral nutrient concentrations under exotic invasive plants in Belgium. Plant Soil 275, 169–179. 10.1007/s11104-005-1257-0

[B46] Vanhellemont M. Wauters L. Baeten L. Bijlsma R. J. De Frenne P. Hermy M. (2010). *Prunus serotina* unleashed: invader dominance after 70 years of forest development. Biol. Invasions 12, 1113–1124. 10.1007/s10530-009-9529-x

[B47] Verheyen K. Vanhellemont M. Stock T. Hermy M. (2007). Predicting patterns of invasion by black cherry (*Prunus serotina* Ehrh.) in Flanders (Belgium) and its impact on the forest understorey community. Divers. Distrib. 13, 487–497. 10.1111/j.1472-4642.2007.00334.x

[B48] Vilà M. Basnou C. Pyšek P. Josefsson M. Genovesi P. Gollasch S. (2010). How well do we understand the impact of alien species on ecosystem services? A pan-European, cross-taxa assessment. Front. Ecol. Environ. 8, 135–144. 10.1890/080083

[B49] Vilà M. Espinar J. L. Hejda M. Hulmpe P. E. Jarošík V. Maron J. L.. (2011). Ecological impacts of invasive alien plants: a meta-analysis of their effects on species, communities and ecosystems. Ecol. Lett. 14, 702–708. 10.1111/j.1461-0248.2011.01628.x 21592274

[B50] Vilà M. Weiner J. (2004). Are invasive plant species better competitors than native plant species? Evidence from pair-wise experiments. Oikos 105, 229–238. 10.1111/j.0030-1299.2004.12682.x

[B51] Wang Q. Zeng Z. Zhong M. (2016). Soil moisture alters the response of soil organic carbon mineralization to litter addition. Ecosystems 19, 450–460. 10.1007/s10021-015-9941-2

[B52] Wayne P. Foster S. Connolly J. Bazzaz F. Epstein P. (2002). Production of allergenic pollen by ragweed (*Ambrosia artemisiifolia* L.) is increased in CO_2_-enriched atmospheres. Ann. Allergy Asthma Immunol. 88, 279–282. 10.1016/S1081-1206(10)62009-1 11926621

[B53] Wright I. J. Reich P. B. Westoby M. Ackerly D. D. Baruch Z. Bongers F.. (2004). The worldwide leaf economic spectrum. Nature 428, 821–827. 10.1038/nature02403 15103368

[B54] Youngentob K. N. Zdenek C. van Gorsel E. (2016). A simple and effective method to collect leaves and seeds from tall trees. Methods Ecol. Evol. 7, 1119–1123. 10.1111/2041-210X.12554

[B55] Zhang L. Ma X. Wang H. Liu S. Siemann E. Zou J. (2016). Soil respiration and litter decomposition increased following perennial forb invasion into an annual grassland. Pedosphere 26, 567–576. 10.1016/S1002-0160(15)60066-2

[B56] Zhang L. Wang H. Zou J. Rogers W. E. Siemann E. (2014). Non-native plant litter enhances soil carbon dioxide emissions in an invaded annual grassland. PLoS ONE 9:e92301. 10.1371/journal.pone.0092301 24647312 PMC3960218

