# Supplementary material for: Invasion by the Alien Tree Prunus serotina Alters Ecosystem Functions in a Temperate Deciduous Forest
Source: Front Plant Sci. 2017 Feb 14;8:179. doi: 10.3389/fpls.2017.00179 (PMC5307375; doi:10.3389/fpls.2017.00179)
Supplement: Supplementary file 2 [file Presentation1.pdf]

Litter decomposition and soil respiration (illustrations)

DIARS-IMG-RA-20150317

Date

17/03/2015

Numbers refer to section numbers in the litter decomposition protocol (DIARS-PR-RA-20150218).

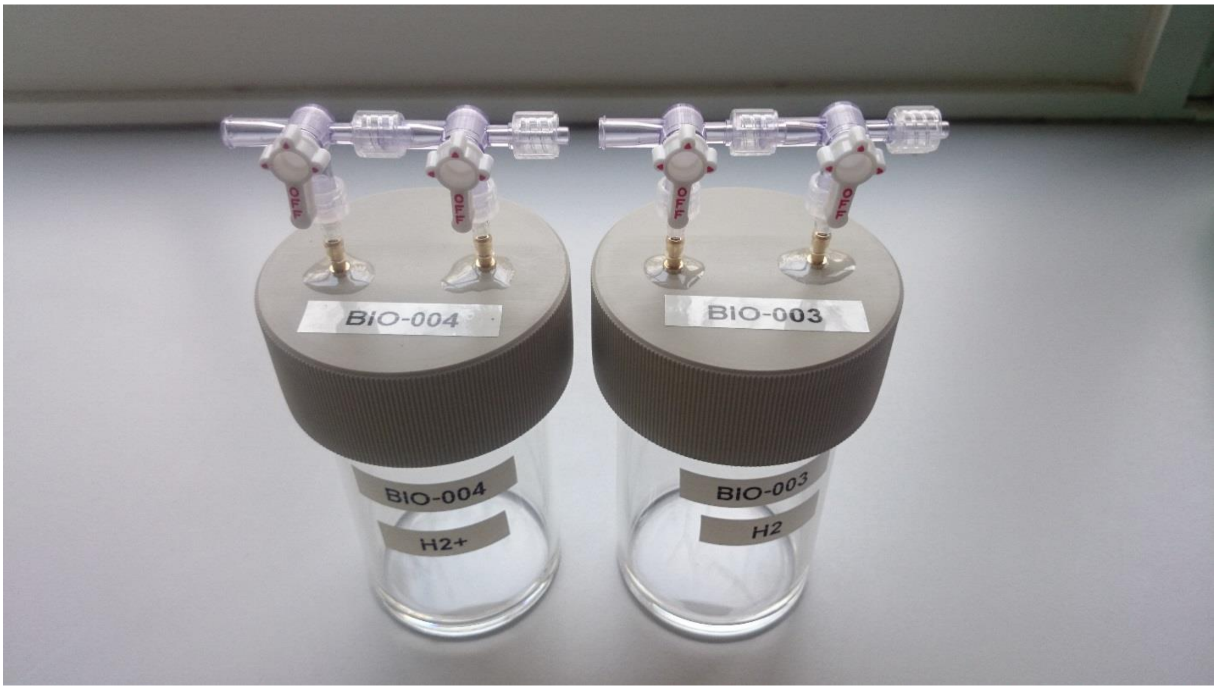

Fig. 1. Air-tight jars fitted with two valves (1.0). A setup with two valves allows the measurement of CO_2 in the jars in a closed circuit. For every plot, one jar will be filled with soil (in this picture, H2), and one jar with soil and litter (here, H2+).

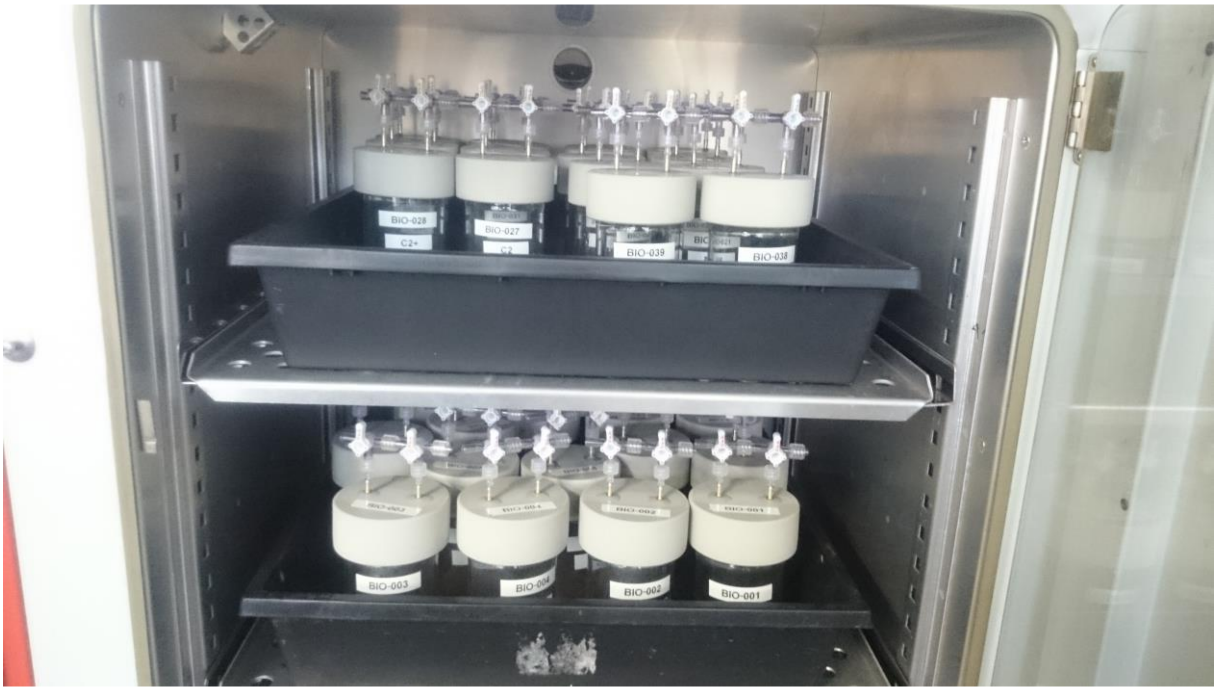

Fig. 2. The jars with moisturized soils (water filled pore space = 60%) are incubated for 12 days at 25°C. Valves are opened (levers are up) (2.6, 2.6.1).

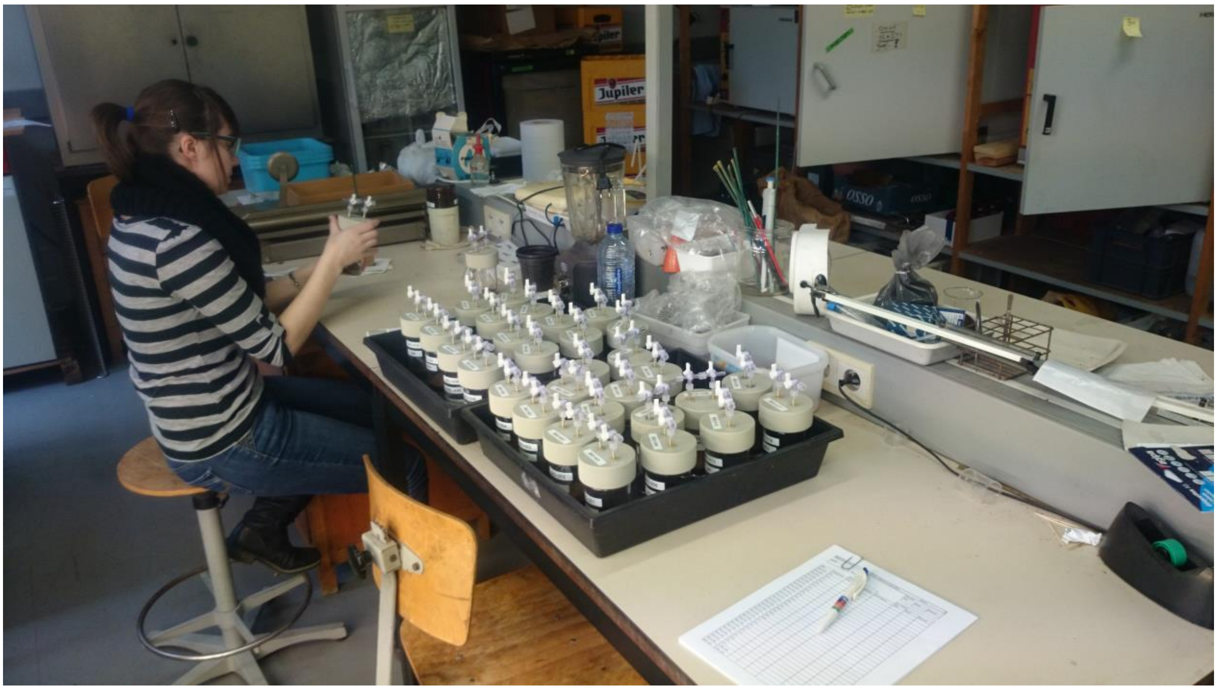

Fig. 3. Ground litter samples are added to the litter jars (+ jars) (3.4).

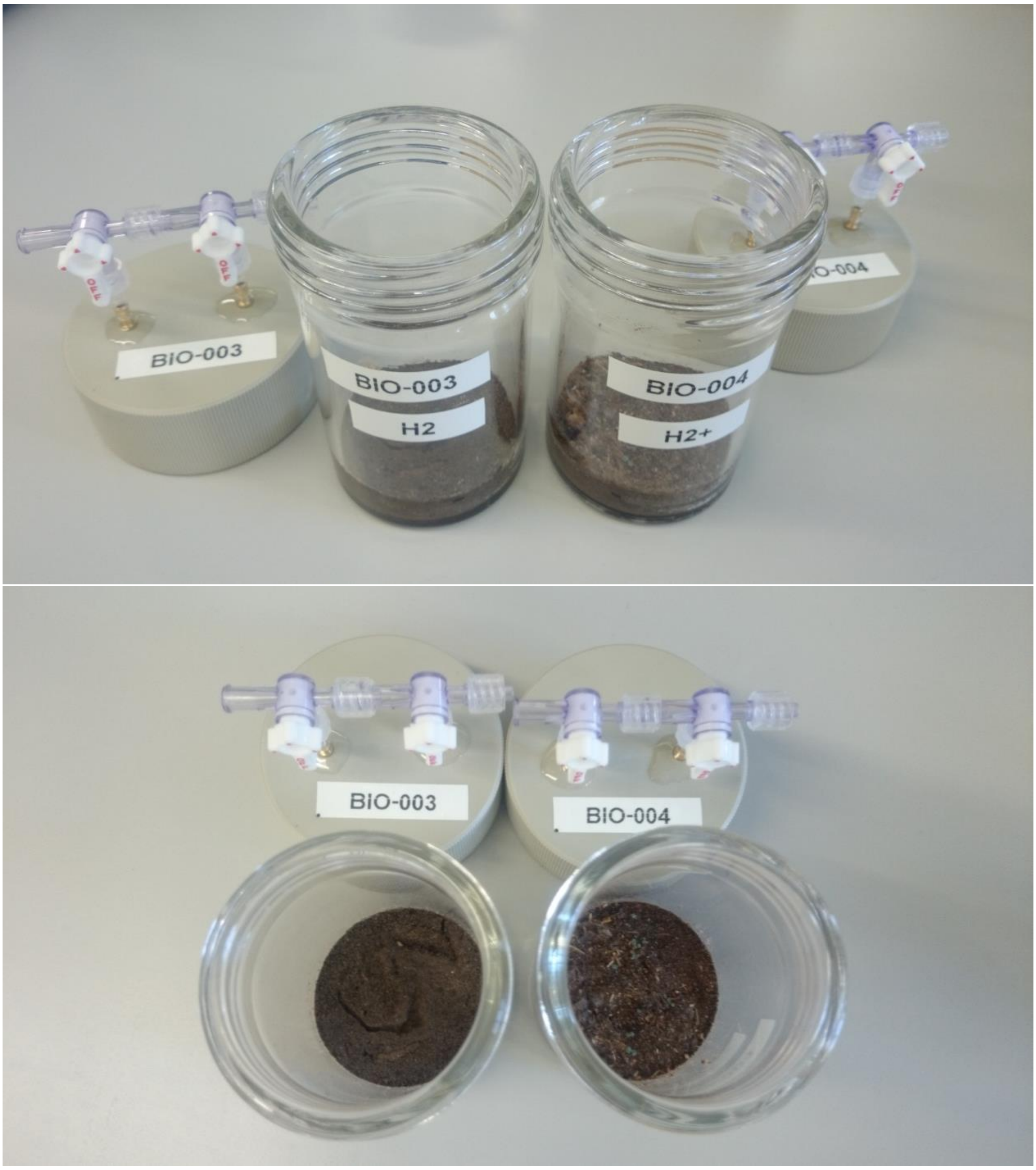

Fig. 4. Jars with experimental soils (40 g of soil, moisturized to a water filled pore space of 60% and set to a bulk density of 1.5 g/cm³) without litter (left) and with litter (right). The amount of litter (mass, in gram per cm² in the jar) is proportional to the available litter in the forest stand (gram per m² in the forest).

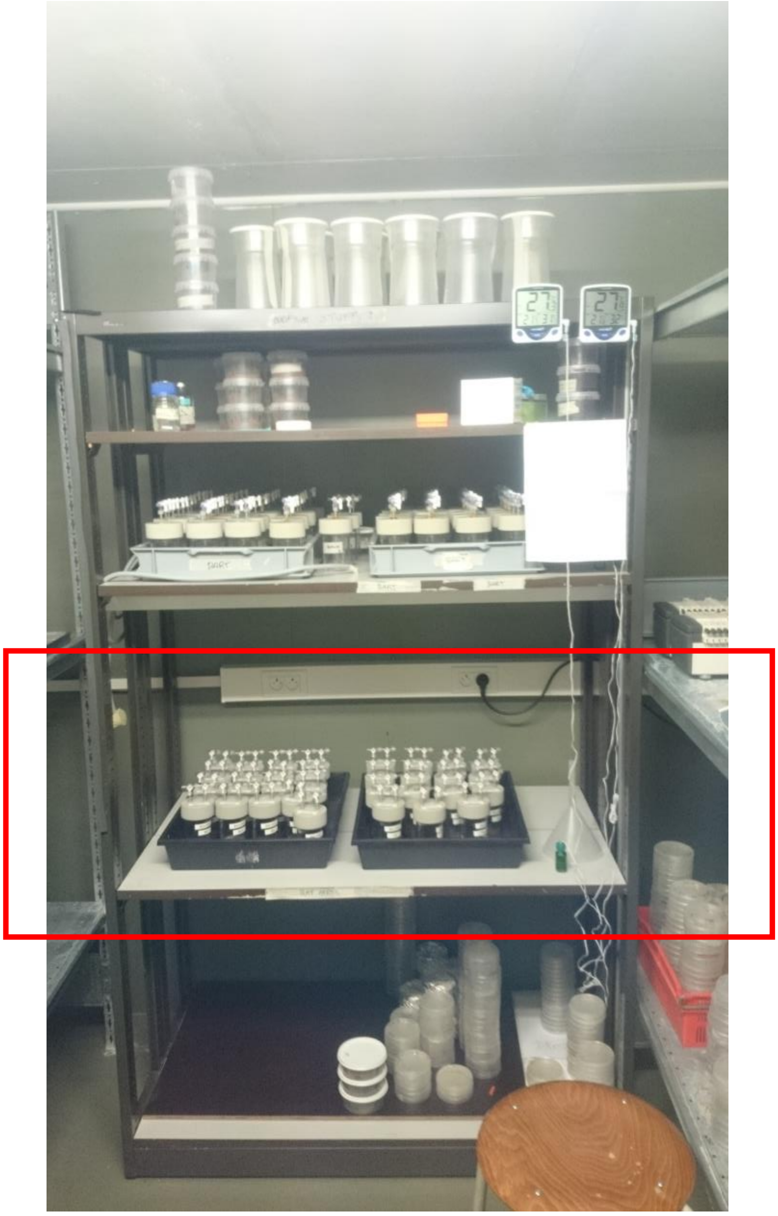

Fig. 5. Jars with closed valves are incubated at 27°C, in a dark room (3.5). Jars are always stored on the same shelf.

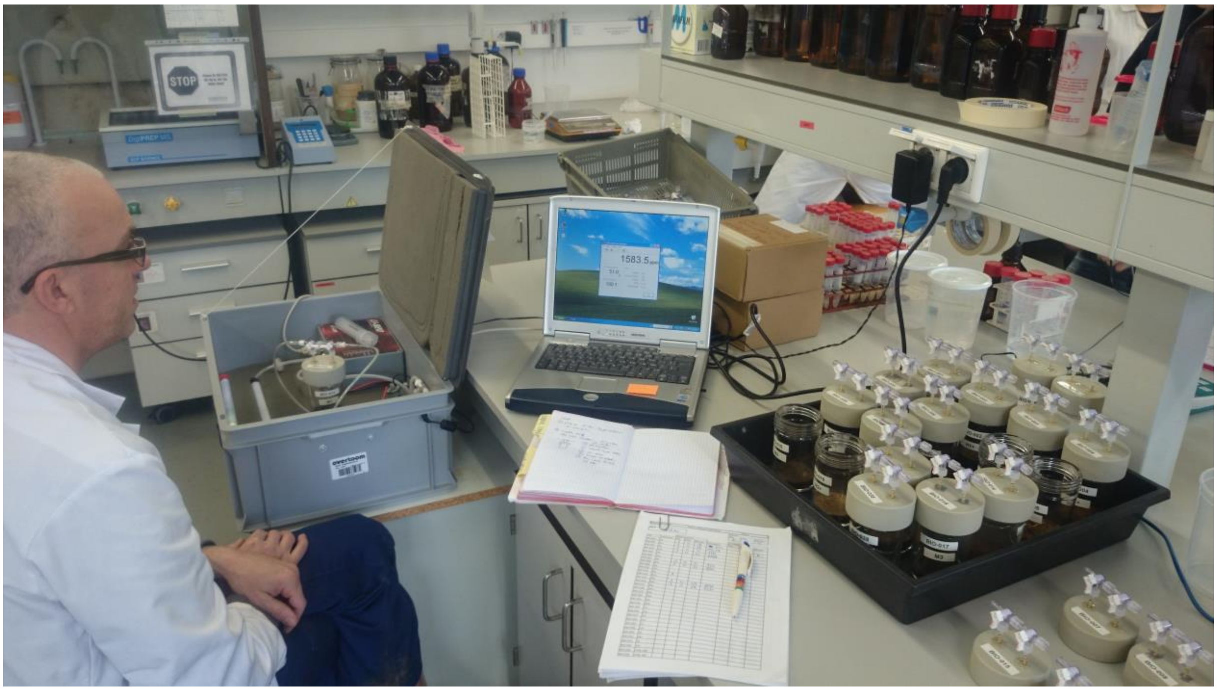

Fig. 6. CO_2 concentrations (in ppm) in the headspace of the jars (from decomposition of soil OM in the soil jars, or from decomposition of litter and soil OM in the + jars) are measured in a closed circuit using a LI-820 CO_2 gas analyser (4.0). Using the ideal gas law, the molecular weight of CO_2 and the dimensions of the jar, CO_2 concentrations in ppm in jar are converted to CO_2 release rates in gram per hectare per hour (g ha^{-1} $hour^{-1}$). The plot level response variables for the DIARS project will be total CO_2 mass released from soil and total CO_2 mass released from soil and litter and these will be calculated by integrating the release rate curves over a set time interval (e.g. three weeks).

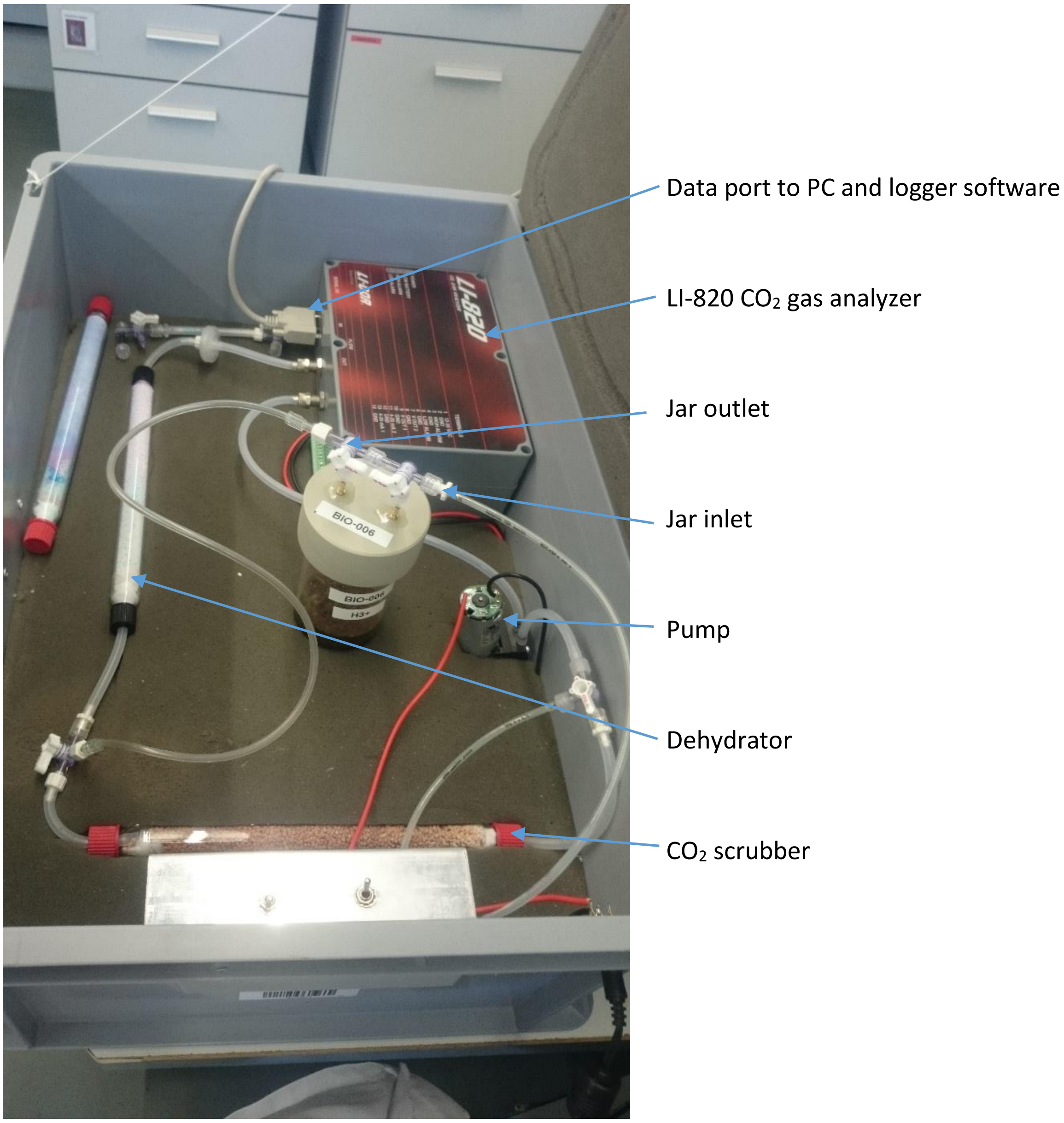

Fig. 7. Closed circuit setup. Jar H3+ is connected in closed circuit to the LI-820 CO_2 gas analyzer. After scrubbing (resetting) the circuit, the jar is connected between outlet and inlet tubes. Scrubber valves are closed (scrubber is taken out of the circuit), jar valves are opened. A pump circulates circuit gas through the jar, leads it through a dehydrator and then to the LI-820 where CO_2 concentration is continuously measured. The pump remains active until a stable concentration is measured, and the final reading is taken after stabilization of cell pressure (100 kPa) and CO_2 concentration value after the pump is switched off. Then, the jar is disconnected, and the circuit is scrubbed (CO_2 removed), first from circuit and jar outlet tube, then from circuit and jar inlet tube.

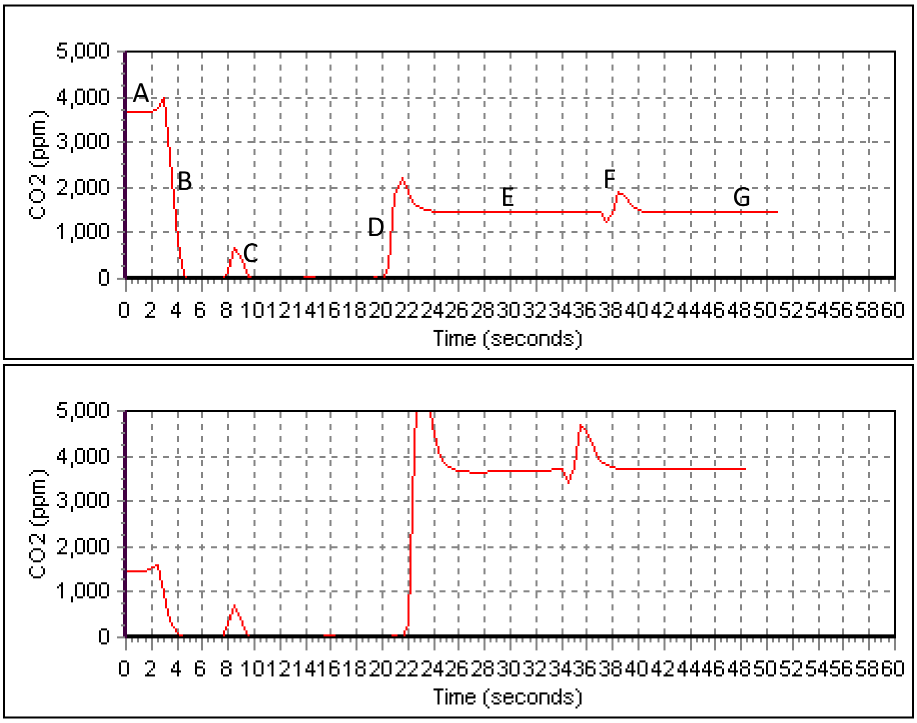

Fig. 8. CO_2 concentration logs of C4 (top) and C4+ (bottom) as measured by the LI820 sensor on 17/3/2015: residual concentration from previous measurement (A), scrubbing circuit and outlet tube (B), scrubbing inlet tube (C), connection of the jar to a CO_2-free circuit and switching pump on (D), reaching stable concentration during pump action (E), pump off (F), and final reading (G) (1446 ppm for C4 and 3710 ppm for C4+).

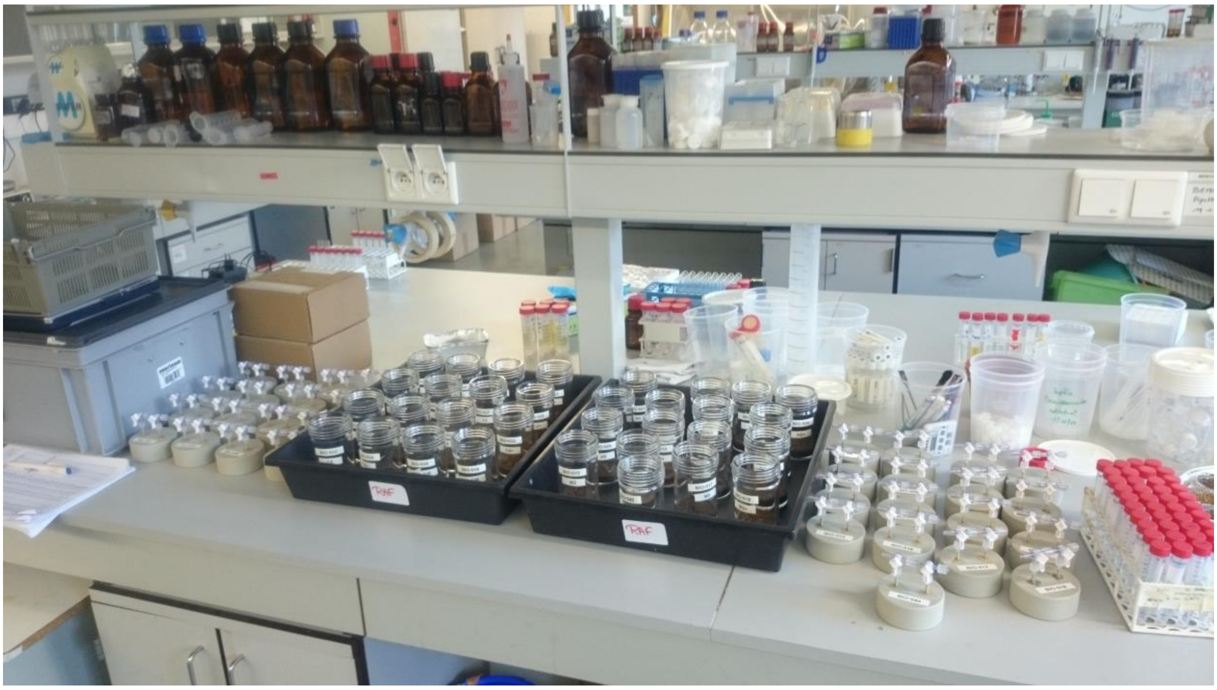

Fig. 9. After each series of measurements, all jars are opened and CO_2 concentrations are allowed to drop to atmospheric concentration. Three blank jars serve as controls for atmospheric CO_2 concentration.

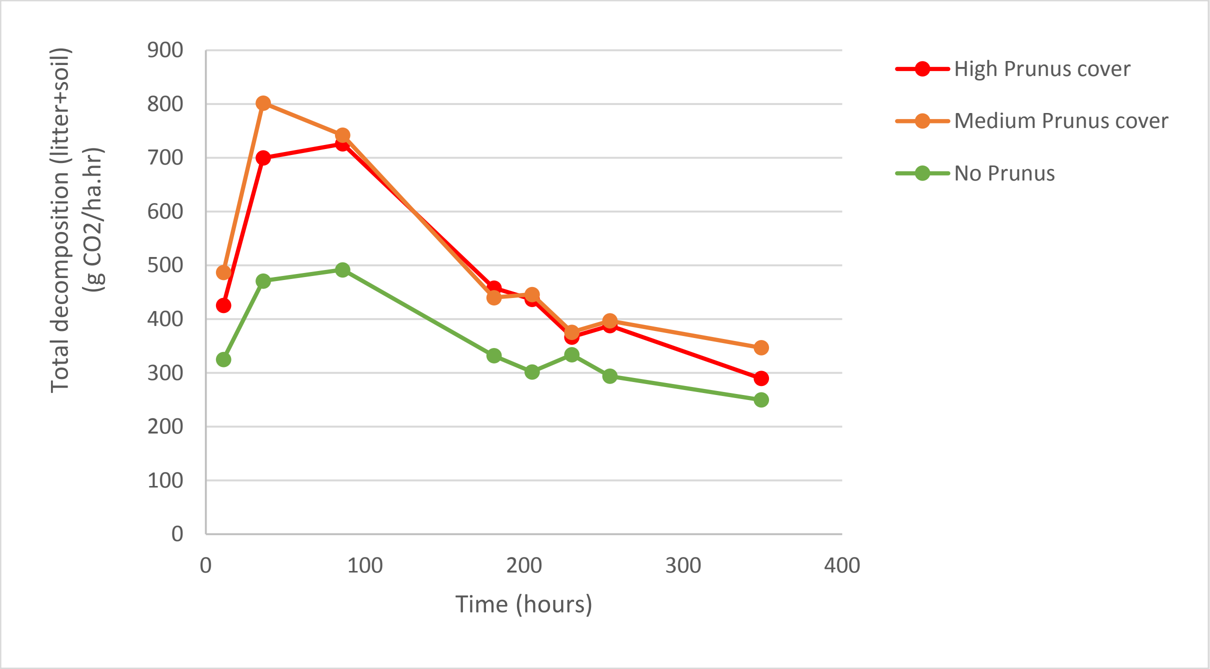

Fig. 10. Organic matter decomposition rate curves (litter + soil OM) showing CO_2 release in gram per ha per hour over 16 days for 15 plots with high, medium or no *Prunus serotina* cover (each *N*=5 plots) in Compiègne forest, France. Standard errors of means overlap and are omitted for clarity.
