# Supplementary material for: Invasion by the Alien Tree Prunus serotina Alters Ecosystem Functions in a Temperate Deciduous Forest
Source: Front Plant Sci. 2017 Feb 14;8:179. doi: 10.3389/fpls.2017.00179 (PMC5307375; doi:10.3389/fpls.2017.00179)
Supplement: Supplementary file 3 [file Presentation2.pdf]

PROTOCOL

Litter and soil respiration

DIARS-PR-RA-20150218

Date

18/02/2015

1. Hardware

1.0. Air-tight jars fitted with two valves (Fig 1).

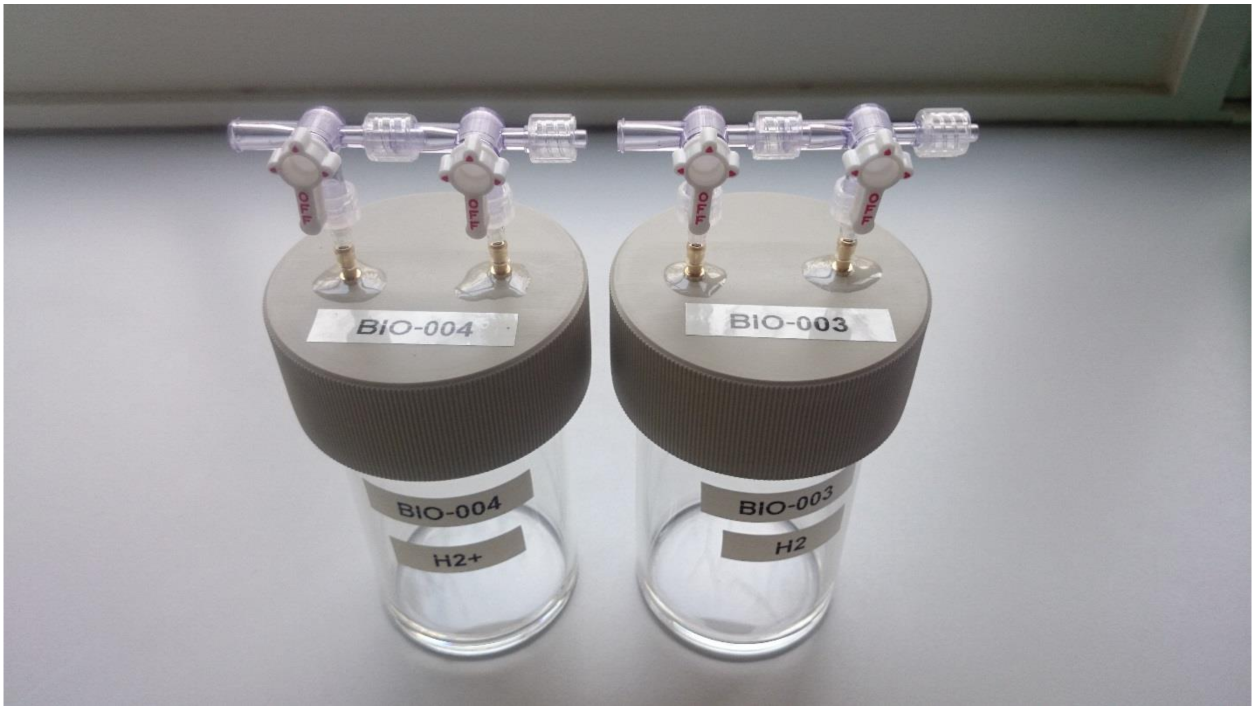

Fig. 1. Air-tight jars for litter decomposition measurement (V=287 ml)

1.0.1. The number of jars needed *Nj = (number of selected plots × 2) + 3*

There are 2 jars per plot: 1 with litter, 1 without litter. There are 4 blanc jars. Two blancs are used to measure atmospheric CO_2 concentrations. Two other blancs are used as references with soil samples.

1.0.2. Before starting the experiment, all jars need to be tested for air-tightness by pressurizing the jars and checking for pressure loss using a manometer.

1.0.3. The exact volume of the jars is to be known. The BIO jars have a volume of **287 ml**.

1.1. Incubation room

1.1.1. The space to store *Nj* jars is a room with ambient room temperature set at 25°C. There is a such a room in the FBIW/EES Soil and Water lab.

1.1.2. The incubation room should be a dark room to prevent algae growth (algae consume CO_2).

1.2. LiCOR820 IR CO_2 sensor

1.2.1. The LiCOR820 IR CO2 sensor should be modified to allow closed-circuit measuring. There is a modified LiCOR CO_2 sensor in the Eric Smolders soil and water lab.

2. Soil preparation

2.0. Soil sample selection

2.1. Soil samples are dried.

2.1.1. Soil samples are first air-dried.
2.1.2. Prior to density measurement and subsampling, soil samples are oven-dried at 50°C for 24 hrs (step added because some samples still felt moist even after months of air-drying).

2.2. Determine air-dry soil sample bulk density and oven-dry organic matter content.

2.2.1. Use dry soil.
2.2.2. Loosely fill container with known volume with dry soil (do not compress). **BD Cup = 73 cm^3**
2.2.3. Weigh soil (in g).
2.2.4. Determine bulk density BD as *weight soil/volume container* (g/cm^3).
2.2.5. Repeat 3 times and calculate average BD.

2.3. Prepare 2 subsamples of a given weight.

2.3.1. The weight of the subsample is determined by the jar dimensions. The compacted soil depth (to standard bulk density of 1.5 g/cm^3) in jar should be 1 cm. Therefore the weight of the subsample is $1.5\ g/cm^3 \times 1\ cm \times S\ cm^2$ g with S the inner bottom surface area of the jar. **BIO jars S = 26.69 cm^2. Subsample weight = 40.04 g**

2.3.2. The two air-dried soil subsamples are transferred to two jars: one sample to jar XN (jar without litter), one sample to jar XN+ (jar with litter)

2.4. Determine soil organic matter content (%)
2.4.1. Determine weight of empty oven cups (one cup per sample) (g)
2.4.2. Fill half of the cup with soil
2.4.3. Oven-dry soil samples (50°C – 24 hours)

2.4.4. Switch on Muffle oven (takes one hour to reach operation temperature)
2.4.5. Determine filled weight of cups (g)
2.4.6. Incubate cups in Muffle oven (3 hours) – **CAUTION – HOT OVEN!**
2.4.7. Remove cups from Muffle oven and let cool down until manageable – **CAUTION – HOT!**
2.4.8. Determine (still warm) weight of cups with heat-treated samples.

2.5. The soils in jars are moisturized to attain a water filled pore space *WFPS* = 60%. Use **demineralized** DEMI water.

2.5.1. The volume of water to be added is calculated as follows:

WFPS = (volumetric water content/soil porosity) x 100 = 60

Soil porosity = 1 – (bulk density/2.65)

Thus: VWC = *0.60 x [1-(bulk density/2.65)]*

volume of water to be added (in ml) = VWC (ml/cm^3) *x soil volume (cm^3)*

Thus: volume of water to be added (in ml) = VWC (ml/cm^3) *x (40 g/ BD (g/cm^3))*

2.6. The jars are incubated for 12 days at 25°C.

2.6.1. Jar valves are open to allow free flux of soil respiration CO_2 to the atmosphere.

2.7. At the end of the incubation period, soil is compacted to standard bulk density of 1.5 g/cm^3.

2.7.1. The standard bulk density is attained by compacting the soil to a set height of 1 cm in jar.

3. Litter preparation

3.0. Oven-dry litter samples

3.1. Mill oven-dry litter samples (using DOMO DO443BL blender) and homogenize

3.1.1. The mill is cleaned after the milling of each sample.

3.2. Milled samples are sieved over a 250 µm mesh (to remove mineral fraction).

3.2.1. The sieve is cleaned with a brush after the sieving of each sample.

3.3. Prepare litter subsamples

3.3.1. The litter subsample should be proportional to the litter quantity in the forest (dry litter weight in g/m^2). As litter samples were collected from $50 \times 50\ cm^2 = 2500\ cm^2$, the quantity of litter to be subsampled is determined by the jar dimensions as follows:
Subsample dry weight = complete sample dry weight × (inside jar bottom surface area S in cm²/2500 cm²) = sample dry weight × (26.69/2500)

3.4. Transfer litter to jar XN+ (jar with litter).

3.4.1. Match and double-check litter sample and soil in jar.

3.4.2. The litter is evenly spread out over the compacted soil in the jar.

3.5. Incubate litter samples.

3.5.1. Jar valves are closed to capture litter decomposition CO_2.

3.5.2. At closing of valves, record decomposition start time

4. Monitor litter decomposition

4.1. Measure and record CO_2 concentration using LiCOR820

4.1.1. The frequency of measurement follows a set monitoring scheme

4.1.2. A. If decomposition is slow (CO_2 concentrations at monitoring moments below 2%), measure in closed circuit following soil and water lab procedure. Chosen method cannot change during experiment.

B. If decomposition is fast (CO_2 concentrations at monitoring moments above 2%), measure in open circuit after dilution with pure O_2 following soil and water lab procedure. Chosen method cannot change during experiment.

4.1.3. At time of CO_2 measurement, record time

4.1.4. After measurement, allow CO_2 concentration to drop to atmospheric background level.

4.1.5. Measure and record atmospheric background level.

4.1.6. Close valves and move jar back to incubation room.

5. Reconstruct CO_2 emission curves

5.1.1. See files DECO-experiment1.xls, DECO-experiment2.xls, DECO-experiment3.xls
