# Supplementary material for: Invasion by the Alien Tree Prunus serotina Alters Ecosystem Functions in a Temperate Deciduous Forest
Source: Front Plant Sci. 2017 Feb 14;8:179. doi: 10.3389/fpls.2017.00179 (PMC5307375; doi:10.3389/fpls.2017.00179)

Supplementary Material

Invasion by the Alien Tree *Prunus serotina* Alters Ecosystem Functions in a Temperate Deciduous Forest

Raf Aerts*, Michael Ewald, Manuel Nicolas, Jérôme Piat, Sandra Skowronek, Jonathan Lenoir, Tarek Hattab, Carol Ximena Garzón-López, Hannes Feilhauer, Sebastian Schmidtlein, Duccio Rocchini, Guillaume Decocq, Ben Somers, Ruben Van De Kerchove, Karolien Denef and Olivier Honnay

*** Correspondence:** Corresponding Author: raf.aerts@kuleuven.be

1 Supplementary Data

The locations of the sample plots are stored in a Google Earth datafile (Google Earth screenshot below).

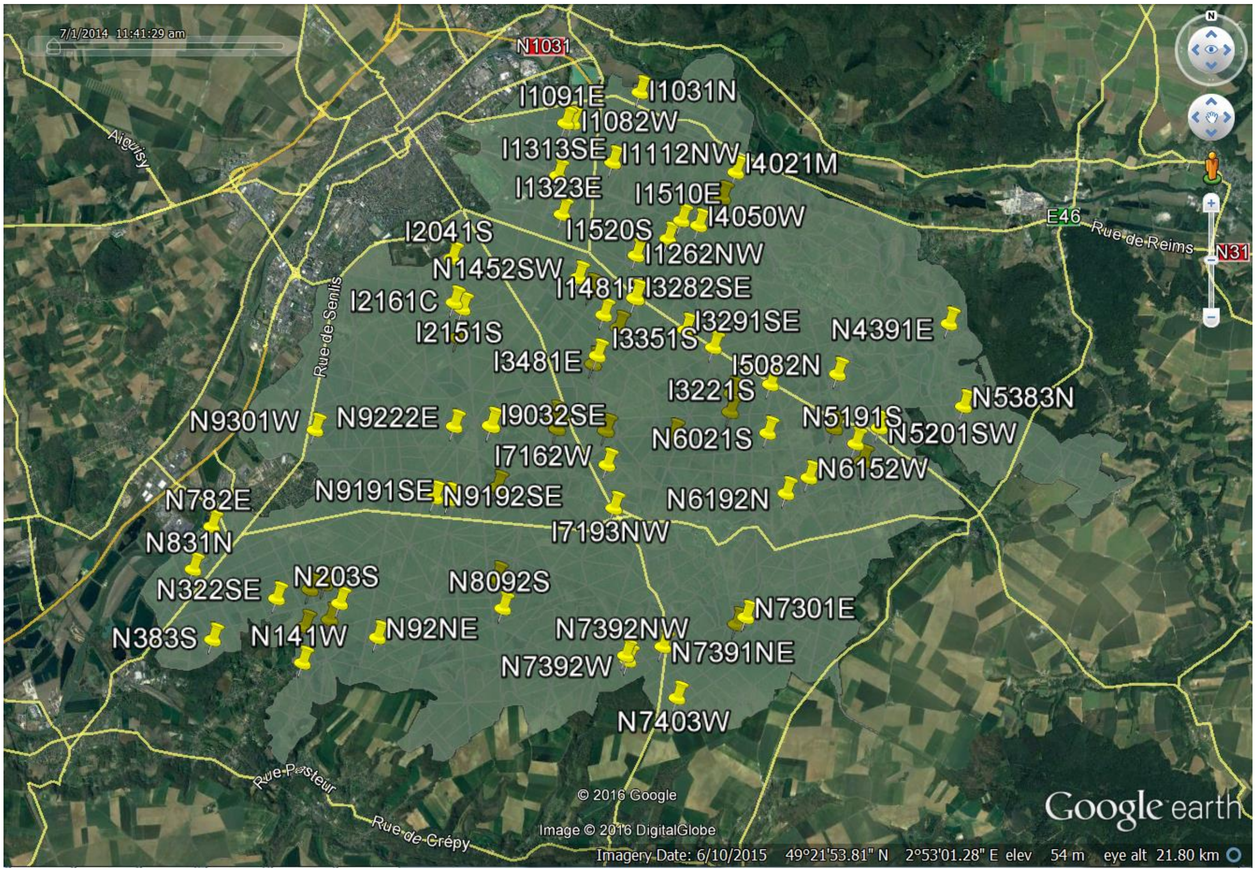

2 Supplementary Tables

Table S1. Reduced General Linear Model for the Heterotrophic Respiration Rate from Soil R_S (kg CO_2 ha^{-1} hr^{-1}) in the Forest of Compiègne, France, with Presence of the Alien Tree American Black Cherry (*Prunus serotina* Ehrh.) (BA > 0.05 m^2 ha^{-1}) as the Fixed Factor, and SOM Content (wt%) and Soil PCA1 (Soil pH and Concentrations of Basic Cations) as Covariates

Source	Type III SS	df	MS	F	P
Corrected model[a]	0.067	5	0.013	14.296	<0.001
Intercept	0.012	1	0.012	13.193	<0.001
Prunus P/A	0.000	1	0.000	0.199	0.658
Soil SOM	0.044	1	0.044	47.267	<0.001
Soil PCA1	0.001	1	0.001	1.401	0.243
Prunus × Soil SOM	0.000	1	0.000	0.075	0.785
Prunus × Soil PCA1	0.000	1	0.000	0.005	0.946
Error	0.039	42	0.001		
Total	0.702	48			

[a] R^2 = 0.630; adjusted R^2 = 0.586

Table S2. Reduced General Linear Model for the Heterotrophic Respiration Rate from Litter and Soil R_{LIT+S} (kg CO_2 ha^{-1} hr^{-1}) in the Forest of Compiègne, France, with Presence of the Alien Tree American Black Cherry (*Prunus serotina* Ehrh.) (BA > 0.05 m^2 ha^{-1}) as the Fixed Factor, and Litter Mass (tonnes ha^{-1}) and Soil PCA1 (Soil pH and Concentrations of Basic Cations) as Covariates

Source	Type III SS	df	MS	F	P
Corrected model[a]	0.315	5	0.063	9.620	<0.001
Intercept	0.193	1	0.193	29.475	<0.001
Prunus P/A	0.035	1	0.035	5.351	0.026
Litter mass	0.154	1	0.154	23.562	<0.001
Soil PCA1	0.061	1	0.061	9.320	0.004
Prunus × Litter mass	0.020	1	0.020	3.091	0.086
Prunus × Soil PCA1	0.012	1	0.012	1.821	0.184
Error	0.275	42	0.007		
Total	5.528	48			

[a] $R^2 = 0.534$; adjusted $R^2 = 0.478$

3 Supplementary Video

Supplementary Video 1. We used Model 55 Goose Guns (Marlin Firearms, Madison, NC) and a fully choked Select Sporting II 12M 12-gauge shotgun (Winchester Repeating Arms, Morgan, UT; featured in this video) with Buckshot 27 ammunition (27 × 6.2 mm pellets) to shoot 325 sets of leaf samples from 307 individual trees in 48 plots. In each plot we aimed to collect leaves from three individuals per species. For broadleaved tree species, one set of 10-15 undamaged leaves was collected from whole branches or twigs shot down from the upper, sun-exposed part of the crown.

The video shows RA (author in image below) selecting a suitable branch and shooting it down using a Winchester shotgun loaded with a single Buckshot 27 cartridge, after which a gloved co-worker emerges from his safety position to catch the descending branch for collecting the leaf samples.

The chokes mounted on the barrels of the shotgun reduce the diameter at the end of the barrel, forcing the pellets in the shot close together, resulting in a tighter shot pattern (for sharp shooting) and increasing effective range (for shooting branches high in the canopy).

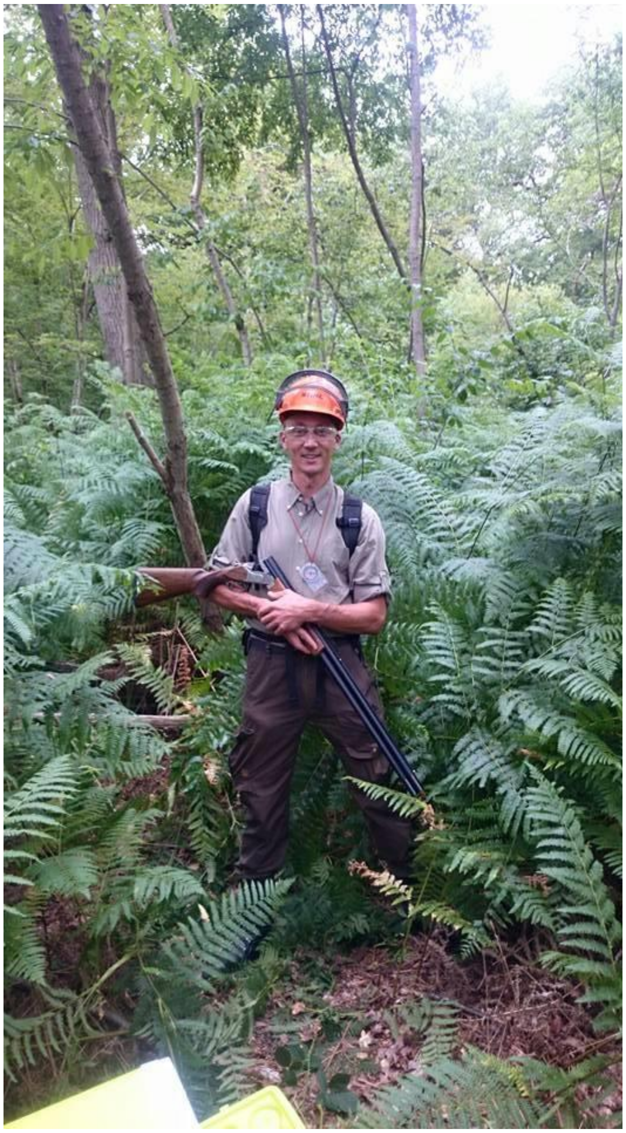

Supplement: Supplementary file 5 [file DataSheet2.pdf]
